# PARP1 negatively regulates MAPK signaling by impairing *BRAF-X1* translation

**DOI:** 10.1186/s13045-023-01428-2

**Published:** 2023-04-03

**Authors:** Andrea Marranci, Antonella Prantera, Simona Masotti, Raffaella De Paolo, Caterina Baldanzi, Maurizio S. Podda, Serena Mero, Marianna Vitiello, Cinzia Franchin, Mariavittoria Laezza, Laura Comelli, Giorgio Arrigoni, Tiziana Cervelli, Giovanna Del Pozzo, Laura Poliseno

**Affiliations:** 1 Institute of Clinical Physiology (IFC), CNR, Via Moruzzi 1, 56124 Pisa, Italy; 2 Oncogenomics Unit, Core Research Laboratory, ISPRO, Via Moruzzi 1, 56124 Pisa, Italy; 3 University of Siena, Siena, Italy; 4 Department of Biomedical Sciences, University of Padova, Padua, Italy; 5 Proteomics Center, University of Padova and Azienda Ospedaliera di Padova, Padua, Italy; 6 Institute of Genetics and Biophysics “Adriano Buzzati Traverso”, CNR, Naples, Italy; 7 Present Address: Fondazione Pisana per la Scienza ONLUS, 56017 Pisa, Italy; 8 Present Address: Genetics, Department of Biology, University of Pisa, 56126 Pisa, Italy; 9 Present Address: Molecular Medicine and Neurobiology, IRCCS Fondazione Stella Maris, 56128 Pisa, Italy

**Keywords:** Melanoma, *BRAF-X1*, mRBP, PARP1, MAPK pathway, Vemurafenib

## Abstract

In human cells *BRAF* oncogene is invariably expressed as a mix of two coding transcripts: *BRAF-ref* and *BRAF-X1*. These two mRNA isoforms, remarkably different in the sequence and length of their *3′UTRs*, are potentially involved in distinct post-transcriptional regulatory circuits. Herein, we identify PARP1 among the mRNA Binding Proteins that specifically target the *X1 3′UTR* in melanoma cells. Mechanistically, PARP1 Zinc Finger domain down-regulates *BRAF* expression at the translational level. As a consequence, it exerts a negative impact on MAPK pathway, and sensitizes melanoma cells to BRAF and MEK inhibitors, both in vitro and in vivo. In summary, our study unveils PARP1 as a negative regulator of the highly oncogenic MAPK pathway in melanoma, through the modulation of *BRAF*-*X1* expression.

**Supplementary Information:**

The online version contains supplementary material available at 10.1186/s13045-023-01428-2.

To the editor

Although BRAFV600E oncogenic kinase is extensively studied as cancer driver and represents a valuable therapeutic target, the regulation of *BRAF* gene expression remains largely unknown [1]. Recently, we reported that, irrespectively of its mutational status, *BRAF* is expressed as a mix of two different splicing variants, namely *BRAF-ref* and *BRAF-X1.* These mRNA isoforms are characterized by *3′UTRs* of different sequence and length (~ 100nt vs. ~ 1300 to 7000nt) [2]. The corresponding protein isoforms differ at the C-terminal, however they are both endowed with kinase activity and together account for BRAFV600E oncogenic features in melanoma cells [2, 3].

A very long *3′UTR* such as the *X1* calls for post-transcriptional regulation. Indeed, we have already identified quite a large group of *X1*-targeting microRNAs that positively or negatively affect RNA stability and translation [4]. Since increasing evidence links RBPs with tumorigenesis [5], we performed a high-throughput screening for *X1*-targeting mRBPs. Such screening led us to the identification of PARP1 as negative regulator of *BRAF-X1* translation and, consequently, of MAPK pathway signaling.

We performed REMSA on the ~ 1300nt long version of *X1 3′UTR*, using S100 cytoplasmic protein extract obtained from A375 melanoma cell line. We observed an *X1*-specific band shift when a radiolabeled riboprobe corresponding to the last 186nt of the *3′UTR* was used (R8 probe in Fig. 1a; see also Additional file 1, Additional file 2: Figs. S1–S3 and Additional file 3: Table S1). Subsequently, we performed a pull-down experiment incubating A375 S100 extract with a desthiobiotinylated R8 riboprobe, and 87 cytoplasmic proteins that specifically bind to the probe were identified by mass spectrometry. Among these proteins, we selected 51 for which no peptides were found in control samples (pull-down performed with a riboprobe of unrelated sequence; see Fig. 1b and Additional file 4: Table S2). STRING analysis revealed that these 51 proteins form a highly interconnected network (Fig. 1c) and belong to pathways related to RNA metabolism (Additional file 5: Table S3). The subset of 20 proteins classified as mRBPs ([6], red nodes in Fig. 1c; see also Additional file 2: Fig. S4) were further characterized using GEPIA database (http://gepia.cancer-pku.cn/detail.php?gene =). 6 mRBPs out of 20 have a prognostic value in melanoma (Additional file 2: Fig. S5), and 5 show a positive correlation with *BRAF* mRNA levels (DHX36, ILF3, KHSRP, PARP1 and STRAP, Additional file 2: Fig. S6). According to two databases (ARED (https://brp.kfshrc.edu.sa/ARED/) and AREsite2 (http://rna.tbi.univie.ac.at/AREsite2/welcome)), the R8 fragment of *X1 3′UTR* does not contain AU-Rich Elements. Therefore, known AUBPs such as ILF3 and KHSRP were not prioritized for further analysis. The binding affinity of the remaining 3 proteins (DHX36, PARP1, and STRAP) with R8 fragment of *X1 3′UTR* was predicted and ranked using the catRAPID omics v2.0 program. As shown in Fig. 1d and Additional file 6: Table S4, the affinity of PARP1 is top-scoring.

**Fig. 1:**
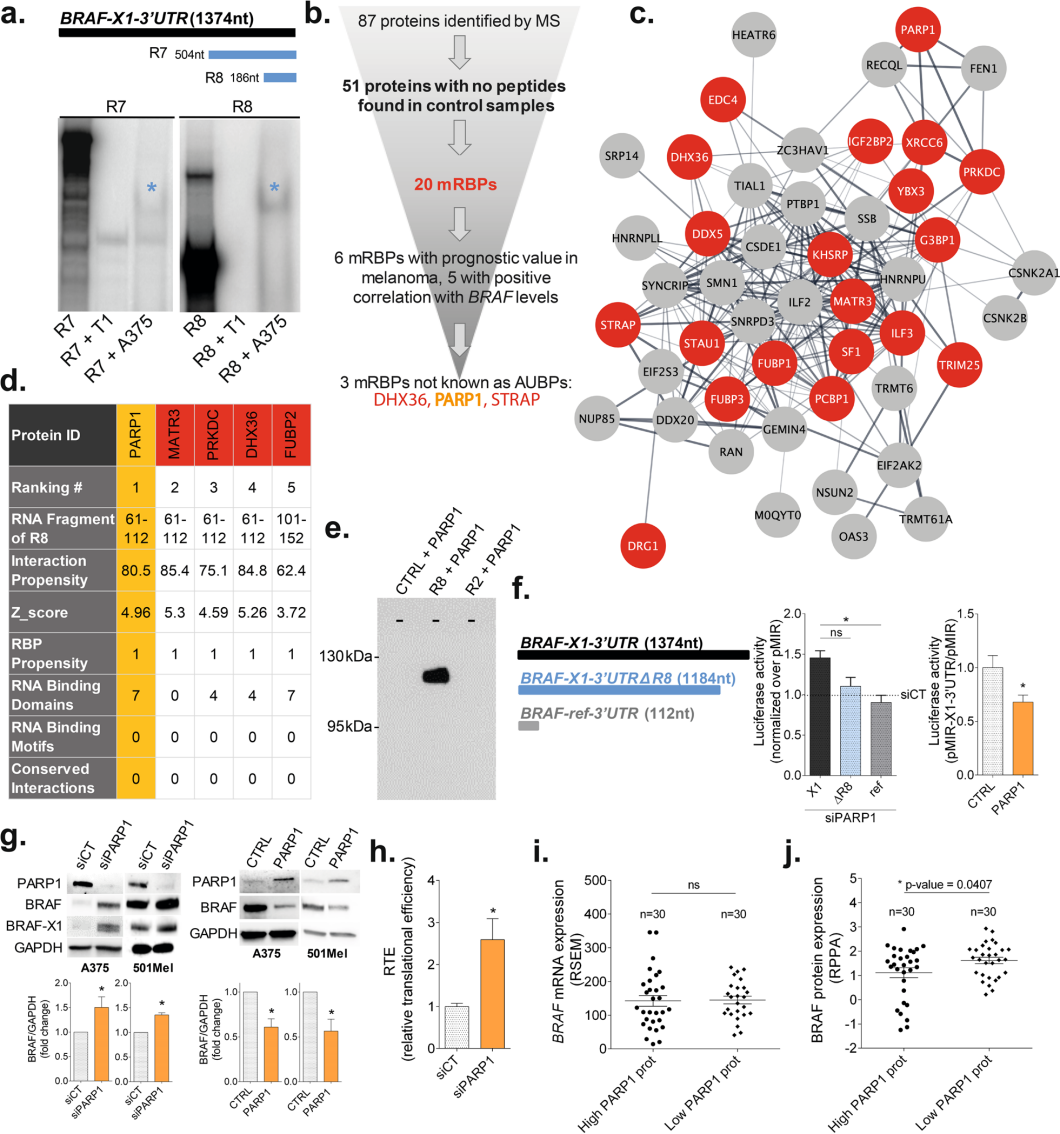
PARP1 directly binds the *3′UTR* of *BRAF-X1* mRNA and negatively regulates its translation in melanoma cells. **a** REMSA. On the top, schematic representation of the radiolabeled riboprobes used for the experiment. On the bottom, band shifts obtained incubating S100 cytoplasmic protein extract obtained from A375 cells with probe R7 and R8 (blue asterisks). See Additional file 2: Fig. S3 for further details. **b** Summary of the analytic workflow to which we subjected the 87 cytoplasmic proteins obtained by mass spectrometry analysis of R8 pull-down. **c** Interaction network of the 51 proteins showing no peptides in pull-down control samples, as obtained by STRING analysis. The 51 proteins are represented as nodes of the network, with the 20 mRBPs highlighted in red. The connections among nodes are depicted as lines and their strength as colors: light gray/gray/black corresponds to 0.5 (weak)/0.75 (intermediate)/1 (strong) overall score. Nodes have been arranged according to the number of connections they have with the other nodes of the network. The lower is the number of connections, the more peripheral is the position of a node. The Protein–Protein Interaction (PPI) enrichment p-value of the network is < 1.0E − 16. **d** Binding affinity of the 20 mRBPs to the R8 fragment of *X1 3′UTR*, according to catRAPID omics v2.0 program. *RNA Fragment of R8*: part of the R8 fragment bound by the mRBP. *Interaction Propensity*: probability of interaction between one protein (or region) and one RNA (or region). *Z_score*: correction of potential biases originating from the length of the RNAs and impacting the Interaction Propensity. *RBP Propensity*: measure of the propensity of the protein to bind the RNA (1 if the protein is in the RBP precompiled library). *RNA Binding Domains*: number of RNA binding domain occurrences found in the protein sequence. *RNA Binding Motifs*: number of RNA binding motif instances found on the RNA sequence. *Conserved Interactions*: number of organisms in which the interaction is conserved out of those in which an orthologous pair is found. *Ranking*: the ranking results from three individual values: (1) catRAPID corrected propensity, (2) RBP propensity, and (3) presence of known RNA Binding Motifs. The 5 top-scoring protein are reported. PARP1 has the highest rank, DHX36 is fourth, while STRAP is 17th (complete list in Additional file 6: Table S4). **e** Pull-down assay reveals the direct binding between desthiobiotinylated R8 riboprobe and recombinant PARP1 protein. Both the unrelated *3′UTR* of Androgen Receptor mRNA, provided by the pull-down kit, and R2 riboprobe, which does not show any band shift when incubated with S100 cytoplasmic protein extract of A375 cells (see also Additional file 1), were used as negative controls. **f** Luciferase assays in A375 cells. The full-length *ref* or *X1 3ʹUTR*, as well as the *X1 3′UTR* missing the R8 region (*X1* ΔR8, left) were cloned downstream of Luciferase CDS in pMIR plasmid, so that pMIR-ref-3′UTR, pMIR-X1-3′UTR and pMIR-X1-3′UTRΔR8 Luciferase plasmids were obtained. Such plasmids were either cotransfected with siCT/siPARP1 in A375 cells (middle), or transfected in A375 cells previously infected with pCW-CTRL/pCW-PARP1 vectors (right). 48 h after transfection, PARP1 knock-down is associated with an increase in the Luciferase activity of pMIR-X1-3′UTR plasmid, but not of pMIR-ref-3′UTR and pMIR-X1-3′UTRΔR8 plasmids, indicating that PARP1 interacts specifically with the *X1 3′UTR*, and more precisely with the R8 region. Consistently, 48 h after transfection and concomitant induction with 2ug/ml of doxycycline, PARP1 overexpression results in a decrease in the Luciferase activity of pMIR-X1-3′UTR plasmid. **g** Western blot analysis of BRAF protein level in A375 and 501Mel cells. Western blot was performed 48 h after the transfection of siPARP1 (left), or 48 h after induction of PARP1 overexpression in cells stably infected with pCW-PARP1 vector and treated with 2ug/ml doxycycline (right). A representative western blot result (top) and bands quantification (bottom) are shown. **h** The RTE of pMIR-X1-3′UTR plasmid, which is the ratio between Luciferase protein activity and Luciferase mRNA level, was calculated in A375 cells, 48 h after the cotransfection of the Luciferase plasmids with siPARP1. Graphs represent the mean ± SEM of at least three independent experiments. *p < 0.05, **p < 0.01, ***p < 0.001, ****p < 0.0001. **i** *BRAF* mRNA expression (RSEM) in the group of high PARP1 protein expressors (25% percentile) versus low PARP1 protein expressors (75% percentile) within the TCGA-SKCM melanoma patient dataset. **j** BRAF protein expression (RPPA) in the group of high PARP1 protein expressors (25% percentile) versus low PARP1 protein expressors (75% percentile) within the TCGA-SKCM melanoma patient dataset

PARP1 has been intensively studied and therapeutically exploited as a nuclear enzyme involved in recognition and repair of DNA damage. However, it has recently emerged as a multifaceted post-transcriptional regulator [7], also considering its partially cytoplasmic localization (Additional file 2: Figs. S7, S8) and its ability to bind mature poly(A) + mRNA [8]. After demonstrating that the binding of PARP1 protein to *X1 3′UTR* is direct (Fig. 1e), we explored the consequences of such binding, in terms of BRAF mRNA/protein levels and MAPK signaling.

Using appropriate Luciferase reporters (Fig. 1f, left and middle) and western blot analysis of A375 (BRAFV600E homozygous) and 501Mel (BRAFV600E heterozygous) melanoma cell lines (Fig. 1g, left), we observed that PARP1 knock down by siRNA leads to an increase in Luciferase activity of full length *X1* reporter, and in endogenous BRAF protein levels, respectively. Conversely, PARP1 stable overexpression by means of inducible pCW-PARP1 vector (Additional file 2: Fig. S9, see also [4]) leads to opposite results (Fig. 1f, right and Fig. 1g, right). Interestingly, in Additional file 2: Fig. S10 we show that the negative regulation exerted by PARP1 on BRAF persists in a context of acquired resistance to vem. Next, we investigated whether PARP1 affects X1 mRNA or protein. We found that PARP1 does not alter *X1* mRNA levels nor stability (Additional file 2: Figs. S11, S12), and rather impairs *X1* mRNA translation (Fig. 1h). Consistently, the K222I mutant, which causes nuclear exclusion (Additional file 2: Fig. S13 and S14a,b), allowed us to confirm that it is cytoplasmic PARP1 to act as negative regulator of BRAF (Additional file 2: Fig. S14c).

To validate our findings using melanoma patient data, we resorted to the TCGA-SKCM dataset. Specifically, we compared *BRAF* mRNA and protein level in the 25% high PARP1 protein expressors versus the 25% low PARP1 protein expressors. Notably, no differences in *BRAF* mRNA level are present between the two groups (Fig. 1i), but the 25% high PARP1 protein expressors show lower BRAF protein levels, in accordance with our in vitro data (Fig. 1j, see also Additional file 2: Fig. S15). All together these data indicate that cytoplasmic PARP1 directly binds to the R8 region and represses *BRAF-X1* translation.

PARP1 consists of three functional domains: the DNA/RNA Binding domain, which in turn is composed of 3 Zinc Finger motifs (Zn), the Auto-modification domain (Auto) and the Catalytic (parylating) domain (Cat) (Fig. 2a). To pinpoint which domain is responsible for *BRAF-X1* regulation, we stably overexpressed each of them separately, by means of inducible pCW-HA vectors (Fig. 2a and Additional file 2: Fig. S13). Interestingly, we found that the Zn domain, which maintains similar intracellular localization as full length PARP1 (Additional file 2: Fig. S16), recapitulates the effect of full length protein in terms of decrease in Luciferase activity (Fig. 2b), decrease in endogenous BRAF protein level (Fig. 2c), and translation impairment (Fig. 2d). We modeled the complex that PARP1 protein forms with R8 RNA fragment and found two interaction sites that fall within the Zn domain (Fig. 2e and Additional file 2: Fig. S17). In addition, the interaction between Zn domain and *X1 3′UTR* was confirmed experimentally, by performing RIP-qRT-PCR analysis on the Zn domain (Fig. 2f). Interestingly, a PARP1 catalytic inhibitor such as Olaparib does not affect endogenous BRAF protein level (Additional file 2: Figs. S18, S19). All together, these results indicate that PARP1 requires the Zn domain to bind *X1* mRNA and that, contrary to what is known so far about PARP1-mediated regulation of mRNA stability [9], parylation is completely dispensable for PARP1-mediated regulation of *X1* mRNA translation.

**Fig. 2:**
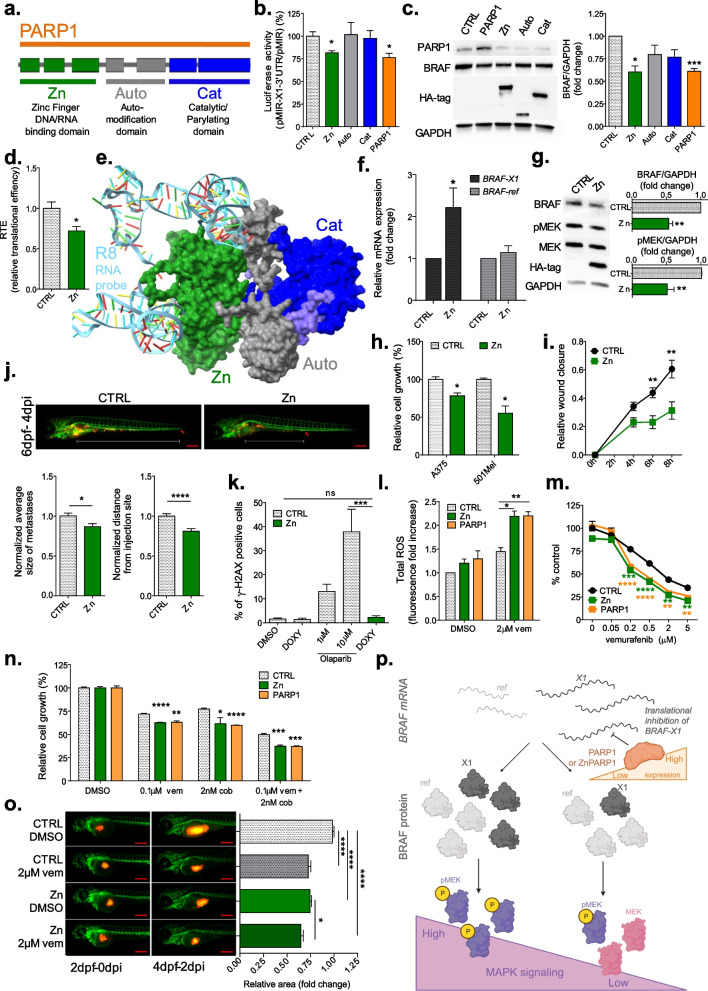
PARP1 Zn domain is responsible for the negative regulation of *BRAF-X1* translation, and inhibits MAPK pathway in vitro and in vivo. **a** Schematic representation of the functional domains of PARP1 protein (3042 bp, 1014 aa): DNA/RNA Binding domain, which in turn is composed of 3 Zinc Finger motifs (Zn, green); Auto-modification domain (Auto, gray); Catalytic (parylating) domain (Cat, blue). **b** Luciferase assay in A375 cells. Among the functional domains tested (pCW-HA-Zn (green), pCW-HA-Auto (gray) and pCW-HA-Cat (blue)), only the overexpression of the Zn domain recapitulates the decrease in Luciferase activity of pMIR-X1-3′UTR plasmid, as observed with full length pCW-PARP1 (orange). The assay was performed 48 h after the transfection of the Luciferase plasmids in cells stably infected with the indicated pCW(-HA) vectors, and the induction of protein overexpression with 2ug/ml doxycycline. **c** Western blot analysis of BRAF protein level in A375 cells stably infected with pCW-CTRL, pCW-HA-Zn, pCW-HA-Auto, pCW-HA-Cat and pCW-PARP1 vectors, 48 h after induction with 2ug/ml doxycycline. A representative western blot result (left) and bands quantification (right) are shown. **d** The RTE of pMIR-X1-3′UTR plasmid was calculated in A375 cells stably infected with pCW-CTRL or pCW-HA-Zn, 48 h after transfection of Luciferase plasmids and induction with 2ug/ml doxycycline. **e** Structural model of PARP1 domain in complex with R8 RNA fragment. PARP1 is represented as *surface*, while R8 is represented as *cartoon*. The Zn domain (residues 1–353) is green, the Auto-modification domain (residues 389–643) is gray and the Catalytic domain (residues 662–1014) is blue. The other residues that do not belong to one of these three domains are purple. R8 phosphate-deoxyribose backbone is cyan, while the color code for nitrogenous bases is as follows: A red; G light green; C yellow; U light blue. **f** RIP-qRT-PCR assay. A375 cells, stably infected with pCW-CTRL or pCW-HA-Zn, were subjected to RIP-qRT-PCR 48 h after induction with 2ug/ml doxycycline. RIP was performed with anti-HA-tag sepharose beads and was coupled with qRT-PCR quantification of *BRAF-ref* and *BRAF-X1* mRNA. **g** Western blot analysis of BRAF and its downstream effector pMEK in A375 cells stably infected with pCW-CTRL or pCW-HA-Zn, 48 h after induction with 2ug/ml doxycycline. A representative western blot result (left) and bands quantification (right) are shown. **h** Proliferation assay of A375 and 501Mel cells stably infected with pCW-CTRL or pCW-HA-Zn, 7 days after induction with 2ug/ml doxycycline. **i** Wound closure assay of A375 cells stably infected with pCW-CTRL or pCW-HA-Zn, 48 h after induction with 2ug/ml doxycycline. **j** Representative pictures (top), size (bottom left) and distance from injection site (bottom right) of metastases developed in a xenograft model in zebrafish embryos. A375 cells, stably infected with pCW-CTRL or pCW-HA-Zn, were resuspended in PBS and were injected in 48hpf embryos. Then, embryos were allowed to grow for 96 h in E3 medium supplemented with 2ug/ml doxycycline. At the end of this period, the size of red cell masses and their distance from injection site were measured. Scale bar: 300um. **k** Percentage of γ-H2AX positive cells. γ-H2AX foci, which mark DNA damage, were stained in A375 cells stably infected with pCW-CTRL or pCW-HA-Zn, 48 h after induction with 2ug/ml doxycycline. pCW-CTRL infected cells were concomitantly treated with the indicated concentrations of Olaparib. See Additional file 2: Fig. S21 for representative pictures of each experimental condition. **l** Total ROS levels measured in A375 cells stably infected with pCW-CTRL, pCW-HA-Zn or pCW-PARP1, after 48 h of induction with 2ug/ml doxycycline and concomitant treatment with 2uM vem. **m** Growth curve of A375 cells stably infected with pCW-CTRL, pCW-HA-Zn or pCW-PARP1, after 7 days of induction with 2ug/ml doxycycline and concomitant treatment with the indicated concentrations of vem. **n** Proliferation assay of A375 cells stably infected with pCW-CTRL, pCW-HA-Zn or pCW-PARP1, 14 days after induction with 2ug/ml doxycycline and treatment with the indicated concentrations of vem, cob or vem + cob. **o** Area of tumors developed in a xenograft model in zebrafish embryos. A375 cells, stably infected with pCW-CTRL or pCW-HA-Zn, were injected in 48hpf embryos. Then, embryos were allowed to grow for 48 h in E3 medium supplemented with 2ug/ml doxycycline and 2uM vem. At the end of this period, the area of red cell masses was quantified. Representative pictures (left) and the results of area quantification (right) are shown. Scale bar: 300um. Graphs represent the mean ± SEM of at least three independent experiments. *p < 0.05, **p < 0.01, ***p < 0.001, ****p < 0.0001. **p** Cartoon summarizing our findings. PARP1 is a new player in the regulation of the highly oncogenic MAPK pathway in melanoma. Through the mRNA binding activity of its Zn domain, it negatively regulates the translation of *BRAF-X1* isoform, leading to a decrease in MAPK signaling and, consequently, a decrease in cell proliferation/motility accompanied by an increase in sensitivity to MAPKi. Cartoon created with BioRender.com

BRAFV600E-X1 downregulation results in a reduction in pMEK, which ultimately affects cellular features such as proliferation and migration [2]. Consistently, we observed that the over-expression of PARP1 Zn domain decreases pMEK levels (Fig. 2g), and impairs cell proliferation (Fig. 2h), as well as cell motility in vitro (Fig. 2i and Additional file 2: Fig. S20) and in a xenograft model in zebrafish (Fig. 2j). Crucially, by detecting no change in γ-H2AX foci, we excluded that Zn domain acts as a dominant negative mutant that is trapped on DNA and triggers DNA damage [7] (Fig. 2k and Additional file 2: Fig. S21). Conversely, in agreement with its role as negative regulator of the MAPK pathway, we found that the Zn domain increases vem-induced ROS (Fig. 2l), hence sensitizes melanoma cells to vem, cob and vem + cob treatment, both in vitro (Fig. 2m, n) and in vivo in a zebrafish xenograft model (Fig. 2o).

In summary, we show that cytoplasmic PARP1 binds *BRAF*-*X1* mRNA and negatively regulates translation (Fig. 2p), in a *3′UTR* binding-dependent, but parylation-independent manner. On one side, our results further consolidate the notion that the X1 variant of *BRAF* is subjected to a tight post-transcriptional regulation, which ultimately has an impact on the output of MAPK pathway. On the other side, our results provide the first example of a specific mRNA whose translation is directly affected by PARP1 mRNA binding activity [10]. Considering that such an mRNA is *BRAF-X1*, they also unveil how PARP1 sphere of influence extends to the regulation of MAPK pathway, hence prompt to explore whether PARP1 is involved in the pathogenesis of those tumor types that are driven by the hyperactivation of such pathway [11]. In more general terms, our data uncover the oncosuppressive liaison existing between *BRAF-X1 3′UTR* and PARP1 Zn domain, challenging the definition of BRAFV600E and PARP1 as pure oncogenes that cooperate in melanocyte transformation [12].

## Supplementary Information

**Additional file 1.** Supplementary Material: REMSA analysis.

**Additional file 2.** Supplementary Figures.

**Additional file 3. Table S1**: Primers.

**Additional file 4. Table S2**: The 87 cytoplasmic proteins identified by mass spectrometry.

**Additional file 5. Table S3**: Reactome pathways to which the 51 selected RBPs belong, listed top to bottom according to increasing FDR values.

**Additional file 6. Table S4**: Binding affinity of the 20 mRBPs to the R8 fragment of X1 3’UTR, according to catRAPID omics v2.0 program.

**Additional file 7.** Material and Methods.

## Data Availability

The datasets generated and/or analyzed during the current study are available from the corresponding author on reasonable request.

## Abbreviations

3′UTR: 3′ UnTranslated Region
AURE: AU-Rich Element
AUBP: AU-Rich Binding Protein
BRAFi: BRAF inhibitors
CDS: CoDing Sequence
cob: Cobimetinib
hpf: Hours post fertilization
MAPKi: Inhibitors of the MAPK pathway
MEKi: MEK inhibitors
mRBP: mRNA Binding Protein
pMEK: Phosphorylated MEK
REMSA: RNA Electrophoretic Mobility Shift Assay
RIP: RNA ImmunoPrecipitation
ROS: Reactive Oxygen Species
RTE: Relative Translational Efficiency
SKCM dataset: SKin Cutaneous Melanoma dataset
TCGA: The Cancer Genome Atlas
vem: Vemurafenib
