# Supplementary material for: PARP1 negatively regulates MAPK signaling by impairing BRAF-X1 translation
Source: J Hematol Oncol. 2023 Apr 3;16:33. doi: 10.1186/s13045-023-01428-2 (PMC10071733; doi:10.1186/s13045-023-01428-2)
Supplement: Supplementary file 1 — Additional file 1. Supplementary Material: REMSA analysis. [file 13045_2023_1428_MOESM1_ESM.docx]

**REMSA analysis**

Different radiolabelled riboprobes, corresponding to sequences downstream of the stop codon, were designed (**Supplementary Fig.1-2**) and used to analyse the binding of S100 cytoplasmic protein extract obtained from A375 cells to *BRAF-ref* *3’UTR* (probe R4) and *BRAF-X1* *3’UTR* (probe R2, 3, 5, 6, 7 and 8).

REMSA results obtained with probe R4 indicate that S100 extract binds to *ref* *3’UTR* (**Supplementary Fig.3**, lane 3).

The binding to *BRAF-X1* *3’UTR* was studied using several radiolabelled riboprobes. No binding was revealed with riboprobes located in the first ~800 nucleotides of the *X1 3’UTR* (probe R2, 3 and 5, data not shown). Conversely, binding was revealed using riboprobes located in the last ~500 nucleotides of the *X1 3’UTR*, namely probe R6 (lane 6), R7 (lane 9 and 14) and R8 (lanes 17, 18, 23 and 30). To determine whether these 3 riboprobes interact with the same or different protein complexes, we performed displacement experiments in presence of cold riboprobes. We observed that cold R6 does not displace R7 (lanes 12 and 13) nor R8 (lanes 21 and 22), whereas cold R8 displaces R7 (lanes 10 and 11) and, conversely, cold R7 displaces R8 (lanes 19 and 20). These results indicate that a protein/protein complex present within A375 S100 cytoplasmic extract binds the very last part of *BRAF-X1* *3’UTR*.

To determine whether the protein/protein complex that binds to R8 is different from the protein/protein complex that binds to R4, we performed further displacement experiments. Since cold R4 does not displace R8 (lane 26-29), we conclude that the R8 fragment of *BRAF-X1* *3’UTR* is bound by different protein/protein complexes compared to *BRAF-ref*.
