# Supplementary material for: PARP1 negatively regulates MAPK signaling by impairing BRAF-X1 translation
Source: J Hematol Oncol. 2023 Apr 3;16:33. doi: 10.1186/s13045-023-01428-2 (PMC10071733; doi:10.1186/s13045-023-01428-2)
Supplement: Supplementary file 2 — Additional file 2. Supplementary Figures. [file 13045_2023_1428_MOESM2_ESM.pdf]

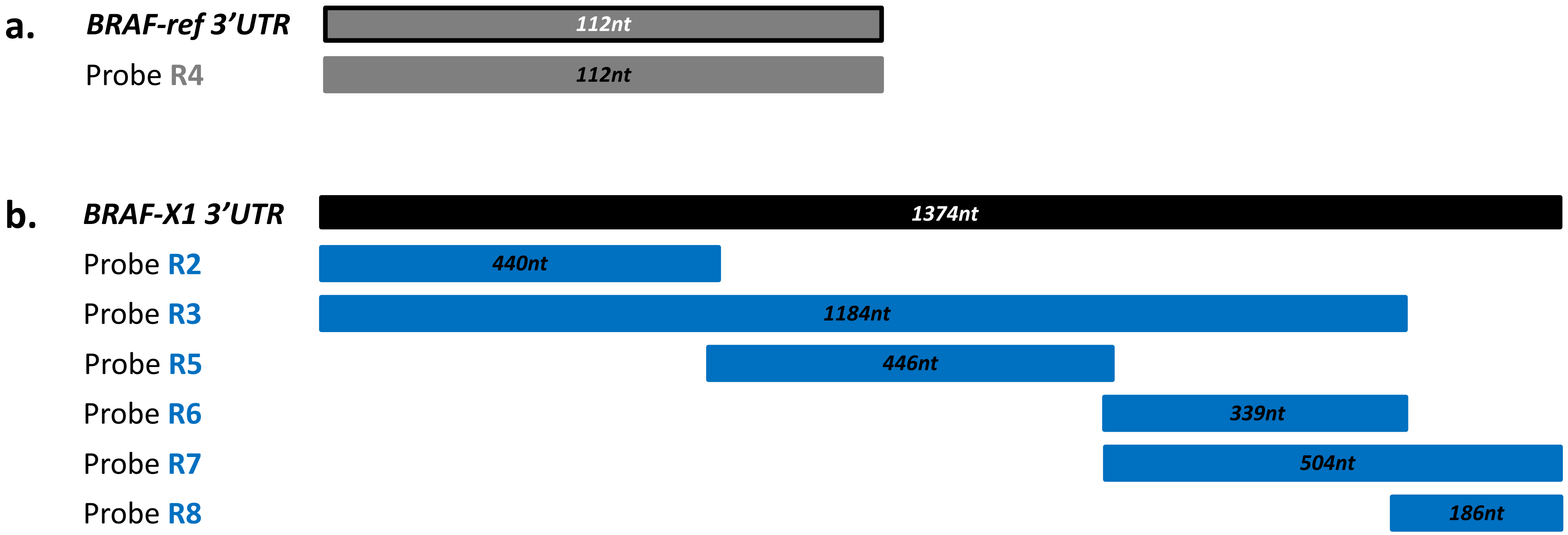

Supplementary Figure 1. Cartoon describing the radiolabeled riboprobes used in REMSA experiments.

ref 3'UTR taken from PMID:25685929

CGCTCATTGCCAAAAATTCACCGCAGTGCATCAGAACCCTCCTTGAATCGGGCTGGTTTCCAAACAGAGGATTTTAG
TCTATATGCTTGTGCTTCTCCAAAAACACCCATCCAGGCAGGGGGATATGGTGCGTTTCCTGTCCAC**TGA**AACAAAT
GAGTGAGAGAGTTCAGGAGAGTAGCAACAAAAGGAAAATAAATGAACATATGTTTGCTTATATGTTAAATTGAATAA
AATACTCTCTTTTTTTTTAAGGTGAACC *AAAAAAAAAAAAAAAAAAAAAAAAA*

TGA-STOP codon

R4 riboprobe – blue, 112nt

X1 3'UTR taken from PMID:25685929

AATTCGATTCGCTCATTGCCAAAAATTCACCGCAGTGCATCAGAACCCTCCTTGAATCGGGCTGGTTTCCAAACAGAGGA
TTTTAGTCTATATGCTTGTGCTTCTCCAAAAACACCCATCCAGGCAGGGGGATATGGAGAATTTGCAGCCTTCAAG**TAG***C*
CACCATCATGGCAGCATCTGCTCTTATTTCTTAAGCCTTGTGTTCGTACAATTTGTTAACATCAAAACACAGTTCTGTTC
CTCAAATCTTTTTTTAAAGATACAAAATTTCCAATGCATAAGCTGATGTGGAACAGAATGGAATTTCCCATCCAACAAAA
GAGGAAAGAATGTTTTAGGAACCAGAATTCTCTGCTGCCAGTGTTTCTTCAACAAAAATACCACGAGCATACAAGTCTGC
CCAGTCCCAGGAAGAAAGAGGAGAGACCCTGAATTCTGACCTTTTGATGGTCAGGCATGATGGAAAGAAACTGCTGCTAC
AGCTTGGGAGATTTGCTATGGAAAGTCTGCCAGTCAACTTTGCCCTTCTAACCACCAGATCAATTTGTGGCTGATCATCT
GATGGGGCAGTTTCAATCACCAAGCATCGTTCTCTTTCCTGTTCTGGAATTTTGTTTTGGAGCTCTTTCCCCTAGTGACC
ACCAGTTAGTTTCTGAGGGATGGAACAAAAATGCAGCTTGCCCTTTCTATGTGGTGCGTGTTCAGGCCTTGACAGATTTT
ATCAAAAGGAAACTATTTTATTTAAATGGAGGCTGAGTGGTGAGTAGATGTGTCTTGGTATGGAGGAAAAGGGCATGCTG
CATCTTCTTCCTGACCTCCGGGGTCTCTGGCCTTTTGTTTCCTTGCTCACTGAGGGGTCTGTCTAACCAAGCAGGCTAGA
TAGTGCTGGCACACATTGCCTTCTTTCTCATTGGGTCCAGCAATGAAGATAAGTGTTTGGGTTTTTTTTTTTCCTCCACA
*ATGTAGCAAATTCTCAGGAAATACAGTTTATATCTTCCTCCTAT****GCTCTTCCAGTCACCAACTACTTATGCGGCTACTTT***
GTCCAGGGCACAAAATGCCGTGGCAGTATCTAACTAAACCCCCACAAAACTGCTTAATAACAGTTTTGAATGTGAGAAAT
TTAGATAATTTAAATATAAGGTACAGGTTTTAATTTCTGAGTTTCTTCTTTTCTATTTTTATTAAAAAGAAAATAATTTT
CAGATTTAATTGAATTGGAAAAAAACAATACTTCCCACCAGAATTATATATCCTGAAAATTGTATTTTTGTTATATAAAC
AACTTTTAAGAAAGATCATTATCCTTTTCTCTACCTAAATATGAGGAGTCTTAGCATAATGACAAATATTTATAATTTTT
CAATTAATGGTACTTGCTGGATCCACACTAACATCTTTGCTAATAATCTCATTGTTTCTTCCAACTGATTCCTAACACTA
TATCCCACATCTTCTTTCTAGTCTTTTATCTAGAATATGCAACCTAAAATAAAAATGGTGGCGTCTCC
AAAAAAAAAAAAAAAAAAAAAA

TAG-STOP codon

R2 riboprobe - blue highlight, 440nt

R3 riboprobe – italics, 1184nt

R5 riboprobe - yellow highlight, 446nt

R6 riboprobe – underlined, 339nt

R7 ripoprobe - bold (**black** and **red**), **504nt**

R8 riboprobe – bold and red, 186nt

region overlapping between R2 riboprobe and R5 riboprobe - green highlight

Supplementary Figure 2. Sequence of the radiolabeled riboprobes used in REMSA experiments.

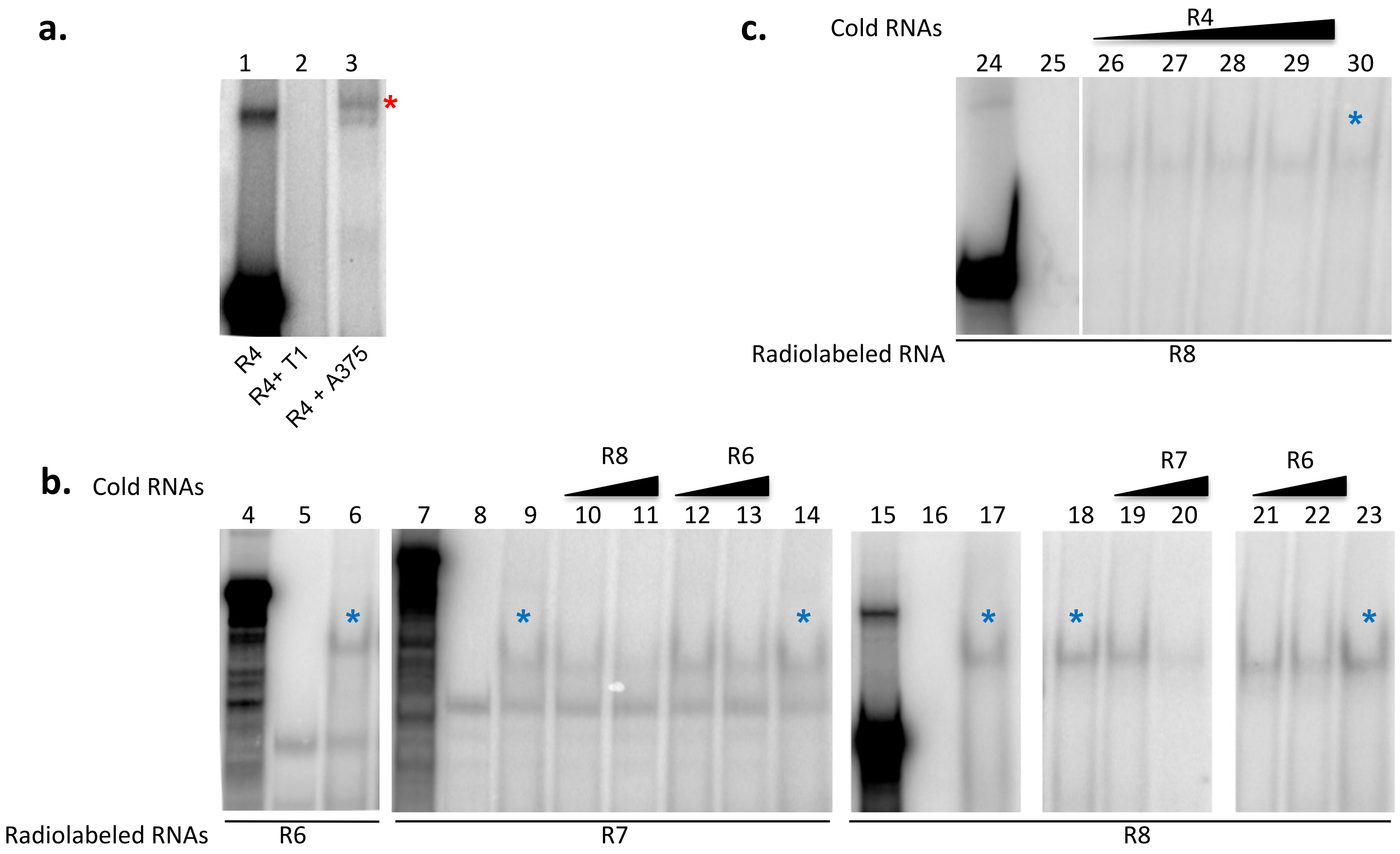

Supplementary Figure 3. REMSA results.

a. The radiolabeled R4 riboprobe for *BRAF-ref 3'UTR* (lane 1) shows a band of interaction with S100 cytoplasmic extract of A375 cells (red asterisk in lane 3); in lane 2 the digestion with T1 RNAse is shown as negative control.

b-c. The radiolabeled riboprobes for *BRAF-X1 3'UTR* (R6 (lane 4), R7 (lane 7) and R8 (lanes 15 and 24)) show a band of interaction with S100 cytoplasmic extract of A375 cells (lanes 6, 9/14 and 17/18/23/30, respectively, blue asterisks); the corresponding RNAse T1 digestions are loaded in lane 5, 8 and 16/25. Radiolabeled R7 is displaced by cold R8 (lanes 10 and 11), while it is not displaced by cold R6 (lanes 12 and 13). Radiolabeled R8 is displaced by cold R7 (lanes 19 and 20), while it is not displaced by cold R6 (lanes 21 and 22), nor by cold R4 (lanes 26-29).

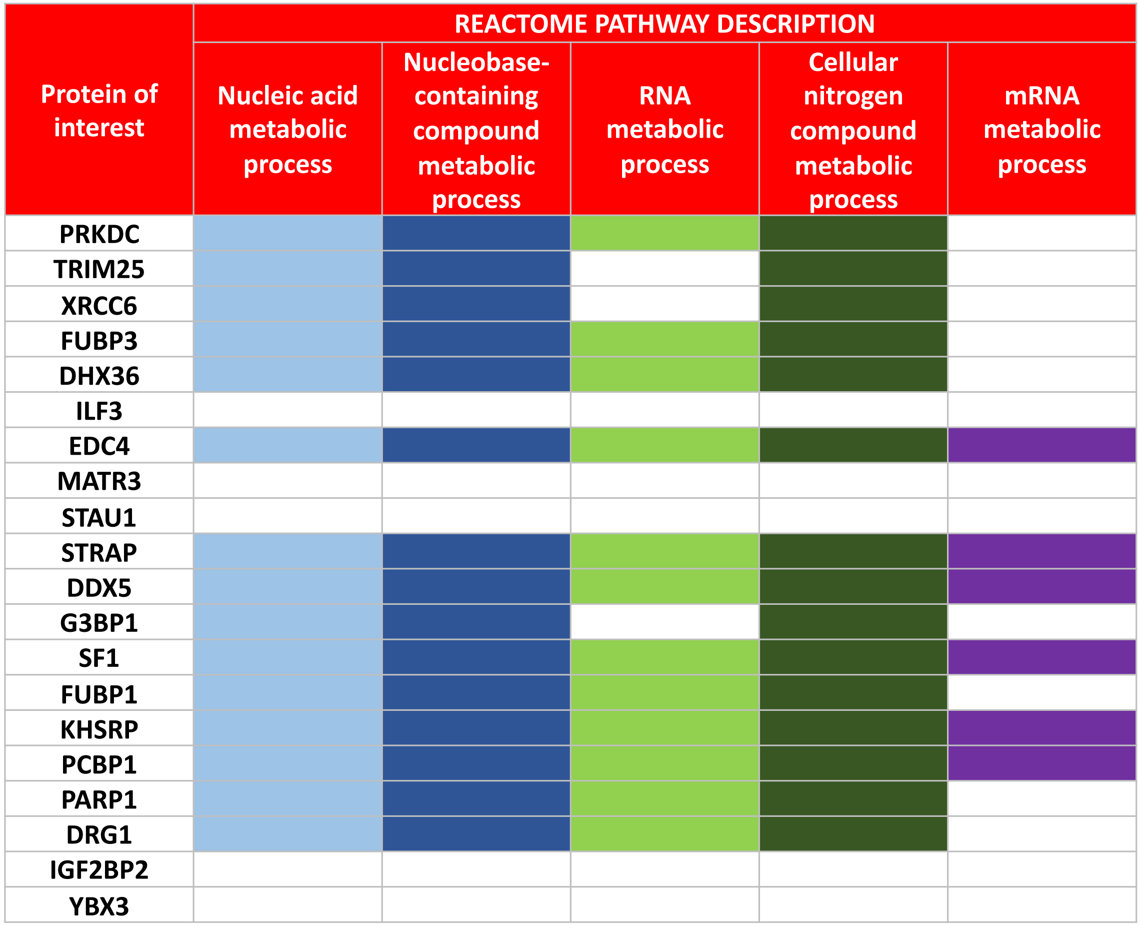

Supplementary Figure 4. Reactome pathway analysis.
The 20mRBPs are reported *(left)*, together with the pathways to which they belong according to Reactome *(right)*. Pathways are listed left to right by increasing FDR values (see also **Supplementary Table 3**).

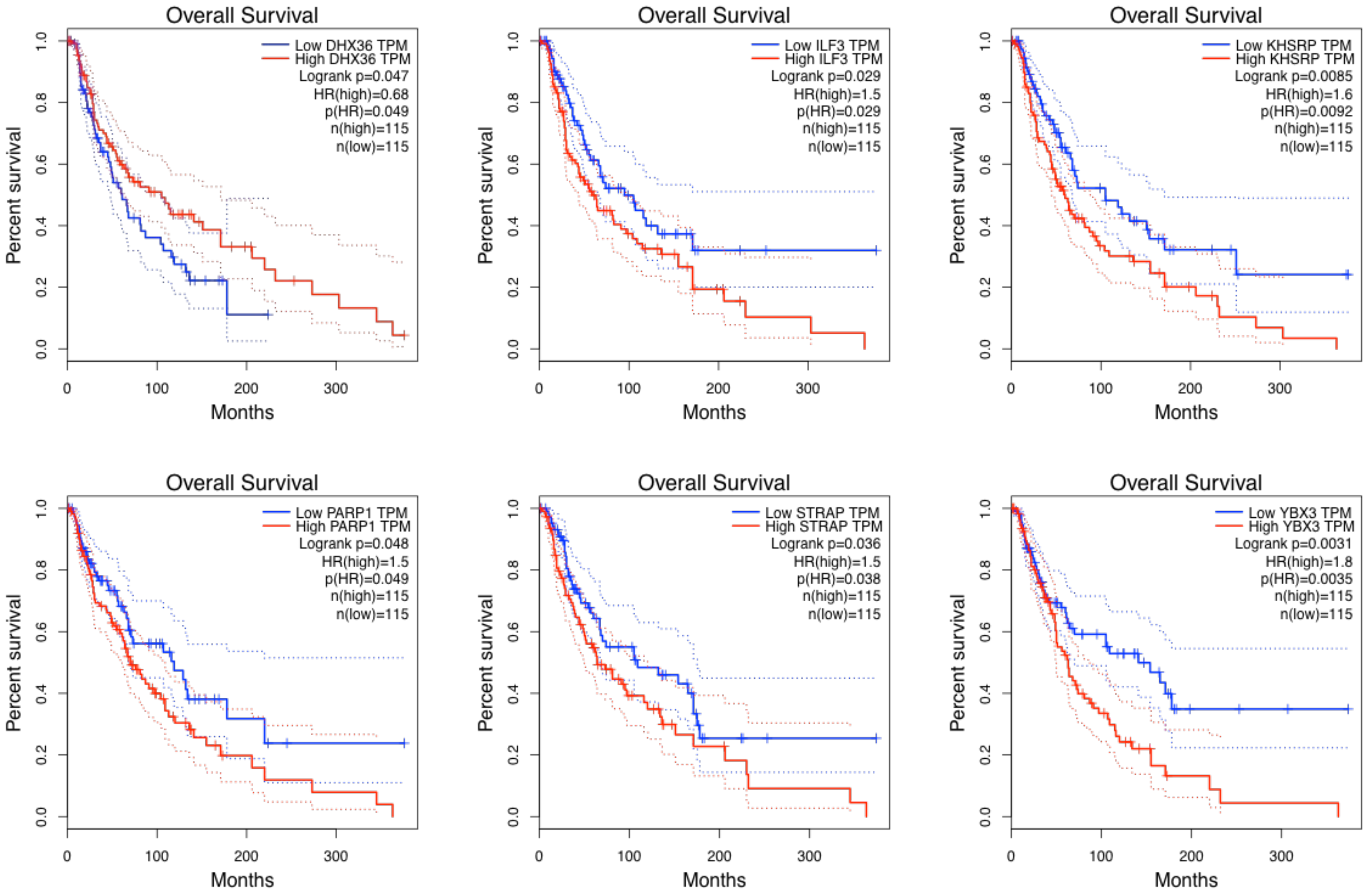

Supplementary Figure 5. mRBPs with prognostic value in melanoma.

The TCGA-SKCM melanoma patient dataset was analyzed by GEPIA (http://gepia.cancer-pku.cn/detail.php).

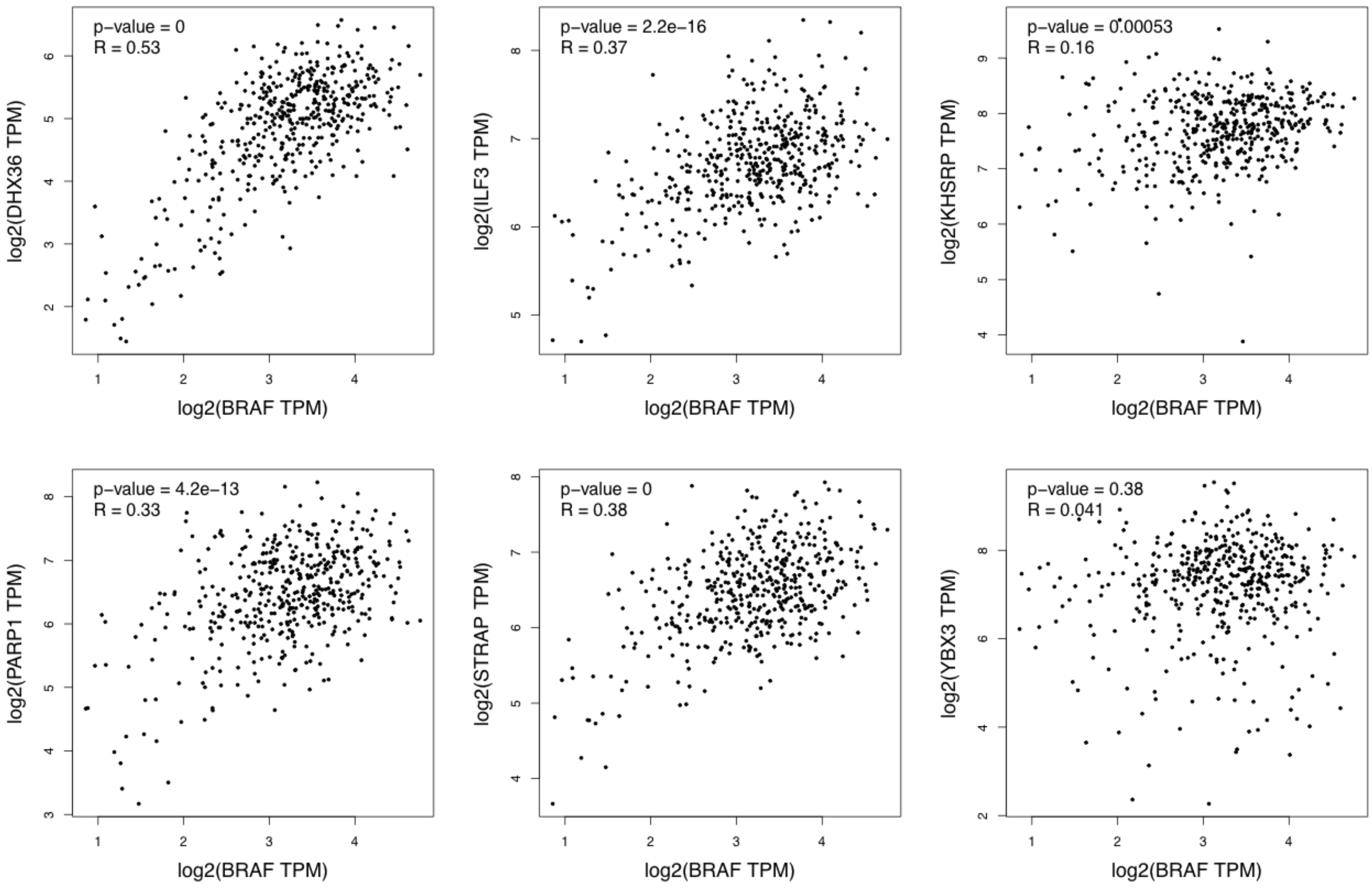

Supplementary Figure 6. Correlation of mRNA levels in melanoma.
The TCGA-SKCM melanoma patient dataset was analyzed by GEPIA (http://gepia.cancer-pku.cn/detail.php).

a.

PARP1 (ENST00000366794.10, *CDS: 172-3216*)

siPARP1_1: pos.822 UG**AAGUGGCGAAGAAGAAAUC**UA

5'-AAGUGGCGAAGAAGAAAUC– dTdT-3'
3’- TdTd-UUCACCGCUUCUUCUUUAG-5'

siPARP1_2: pos.2659 GA**CUUGGAAGUCAUCGAUAUC**UU

5'-CUUGGAAGUCAUCGAUAUC-dTdT-3'
3’-TdTd-GAACCUUCAGUAGCUAUAG-5'

b.

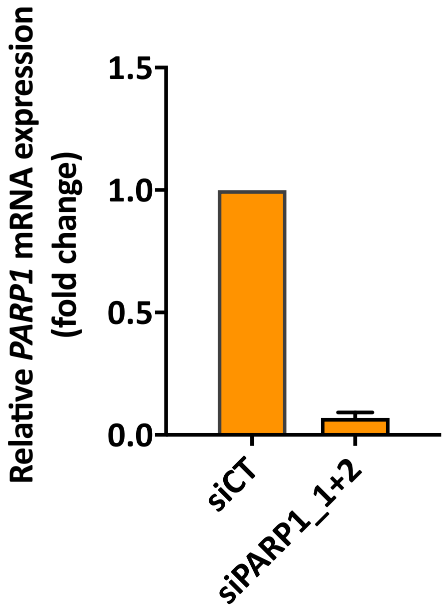

Supplementary Figure 7. siRNAs against PARP1.

a. Sequence and target sequence of siPARP1_1 and siPARP1_2, the 2 siRNAs designed to downregulate PARP1 expression.

b. The efficacy of the siRNA mix (siPARP1_1 plus siPARP1_2, in 1-to-1 ratio) in downregulating PARP1 expression was assessed by qRT-PCR at 48h after transient transfection in A375 cells.

Throughout the article, the 2 siRNAs have always been used together as a 1-to-1 mix and referred to as siPARP1.

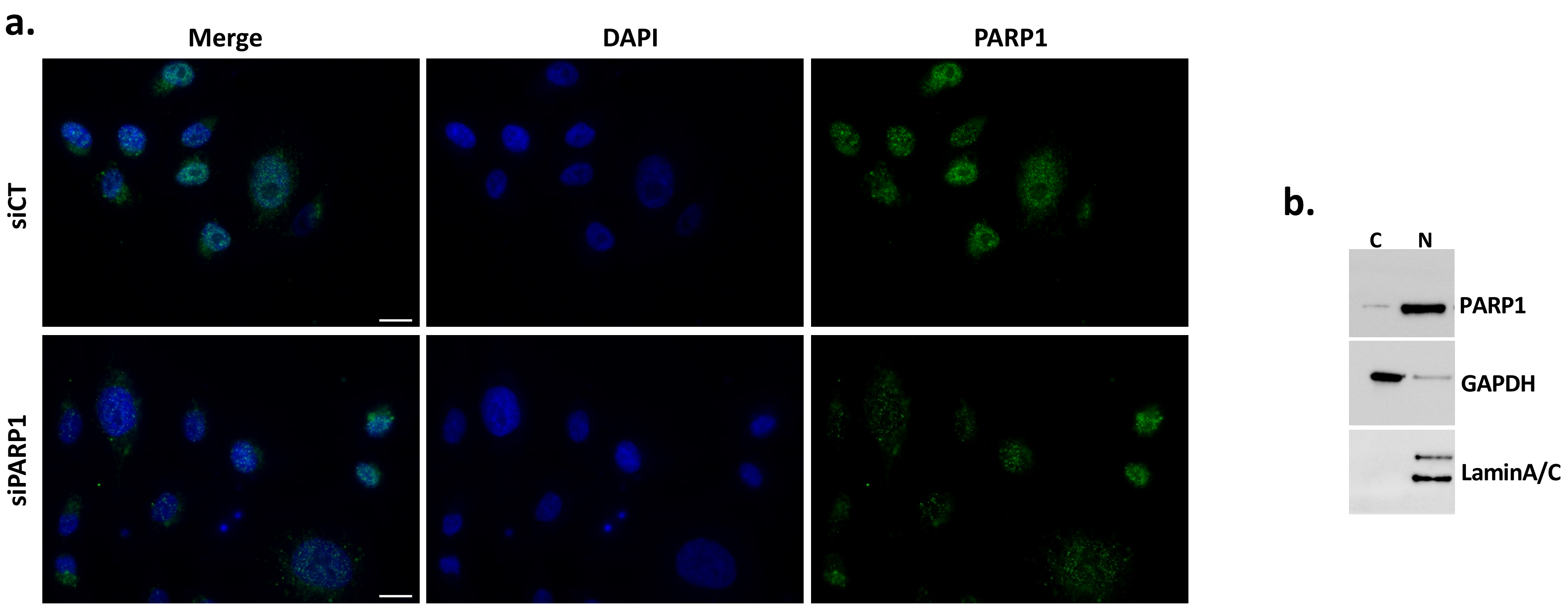

Supplementary Figure 8. Localization of endogenous PARP1.

a. Endogenous PARP1 detected by immunofluorescence in A375 cells, 48h after transient transfection of siCT *(upper)* or siPARP1 *(lower)*. Blue: DAPI; green: PARP1. Scale bar: 10um.

b. Western blot detection of PARP1 in the cytoplasmic (C) and nuclear (N) fractions obtained from A375 cells. GAPDH and LaminA/C are included as positive controls for the cytoplasmic and nuclear fraction, respectively.

PARP1 localizes mainly in the nucleus, but also in the cytoplasm (see also PMID: 30614530 and PMID: 35460603).

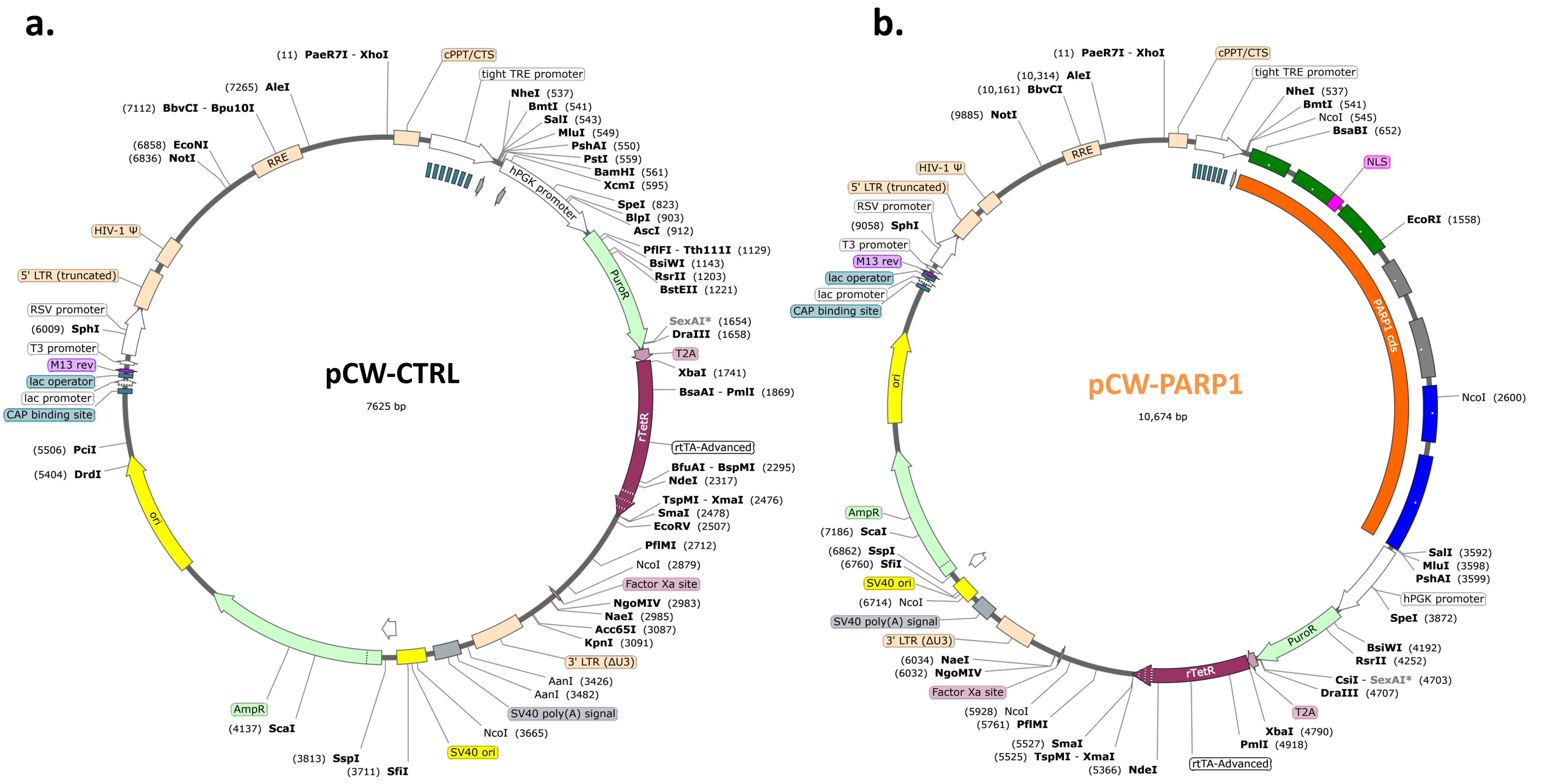

Supplementary Figure 9. Map of pCW vector used to overexpress full length PARP1.

a. Map of pCW empty vector, used as backbone for the cloning of full length PARP1, and as negative control (pCW-CTRL) in all infection experiments.

b. Map of pCW-PARP1 vector.

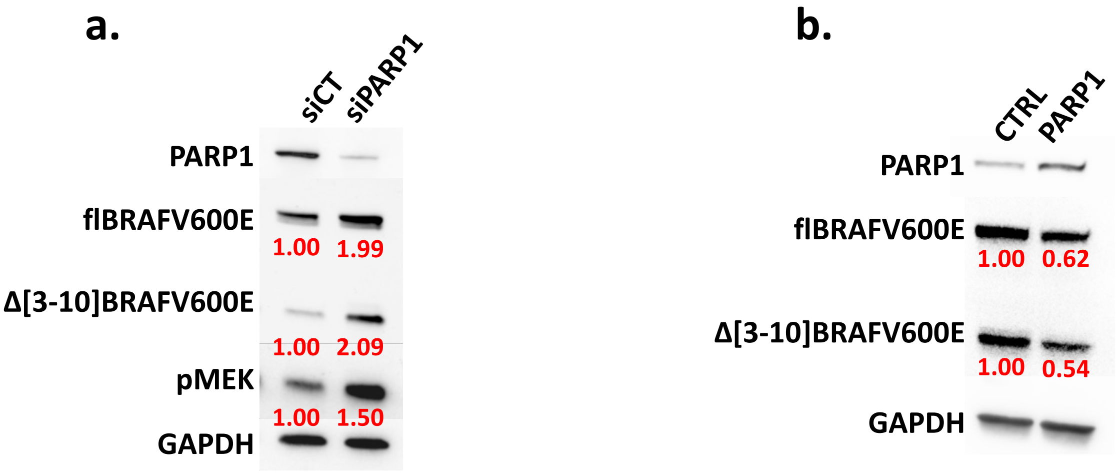

Supplementary Figure 10. Western blot analysis of BRAFV600E protein level in A375 C2 vem-resistant clone.

As reported in PMID: 28445987, A375 C2 vem-resistant clone derives from parental A375 cells and expresses the vem-insensitive Δ[3-10] splicing variant of BRAFV600E. As reported in PMID: 28454577, A375 parental cells and A375 C2 vem-resistant clone share the same composition in *BRAF* isoforms.

a. Representative western blot performed on A375 C2 vem-resistant cells 48h after transient transfection of siPARP1 shows an increase in both full length and Δ[3-10] BRAFV600E level, and a concomitant increase in pMEK level. Quantification of band intensity (fold change over siCT control) is reported in red.

b. Representative western blot performed on A375 C2 vem-resistant cells, stably infected with pCW-CTRL or pCW-PARP1 vectors, 48h after induction with 2ug/ml doxycycline. A decrease in both full length and Δ[3-10] BRAFV600E level is shown. Quantification of band intensity (fold change over pCW-CTRL) is reported in red.

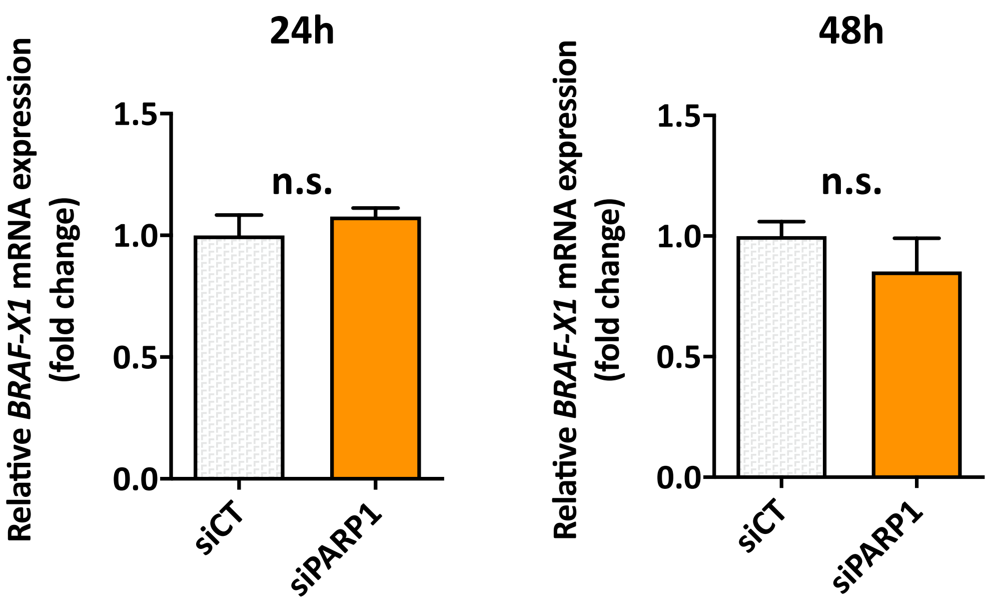

Supplementary Figure 11. ***BRAF-X1*** **mRNA level measured by qRT-PCR 24h** ***(left)*** **and 48h** ***(right)*** **after transient siPARP1 transfection.**
Graphs represent the mean±SEM of at least three independent experiments.

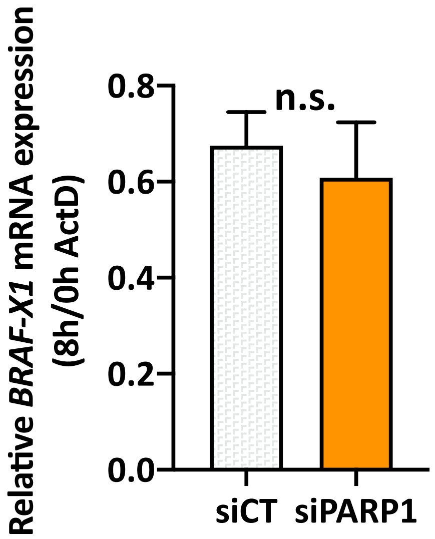

Supplementary Figure 12. Actinomycin D treatment.

Real-time PCR quantification of *BRAF-X1* mRNA in A375 cells that were transfected with siPARP1 and 24h later treated with 10ug/ml Actinomycin D (ActD) for 8h. The graph represents the mean±SEM of at least three independent experiments.

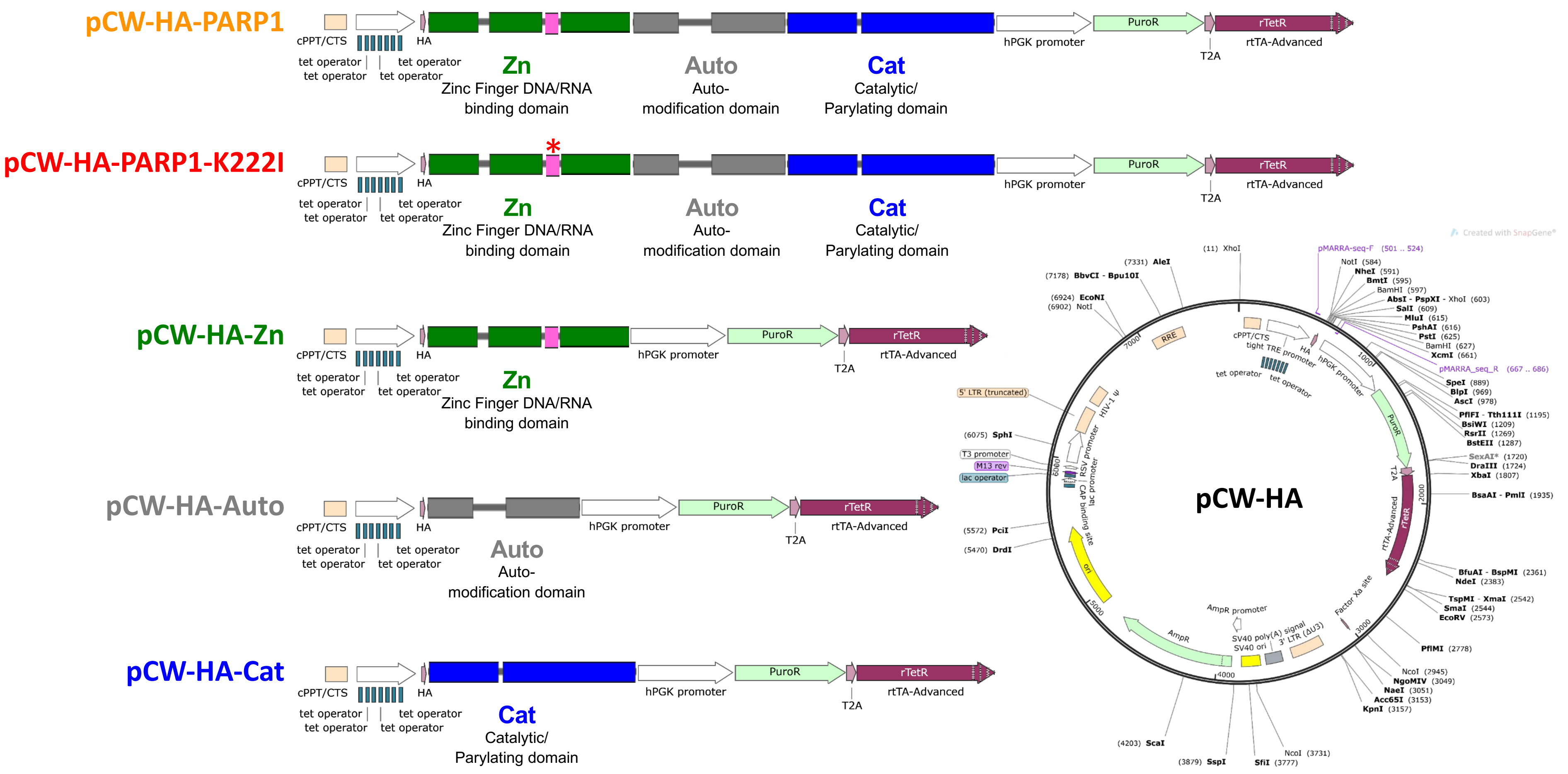

Supplementary Figure 13. Map of pCW-HA vectors used to overexpress full length PARP1, mutant PARP1-K222I, and PARP1 domains.
Orange: full length PARP1.
Red: mutant PARP1-K222I (see **Supplementary Fig.14**).
Green: Zinc Finger DNA/RNA binding domain. The 3 Zinc finger (Zn) motifs are represented as green boxes, while the Nuclear Localization Signal (NLS), located between the second and the third Zn motif, is represented as pink box. The K222I mutation withing the NLS is represented as a red asterisk.
Gray: Auto-modification domain (Auto).
Blue: Catalytic/Parylating domain (Cat).

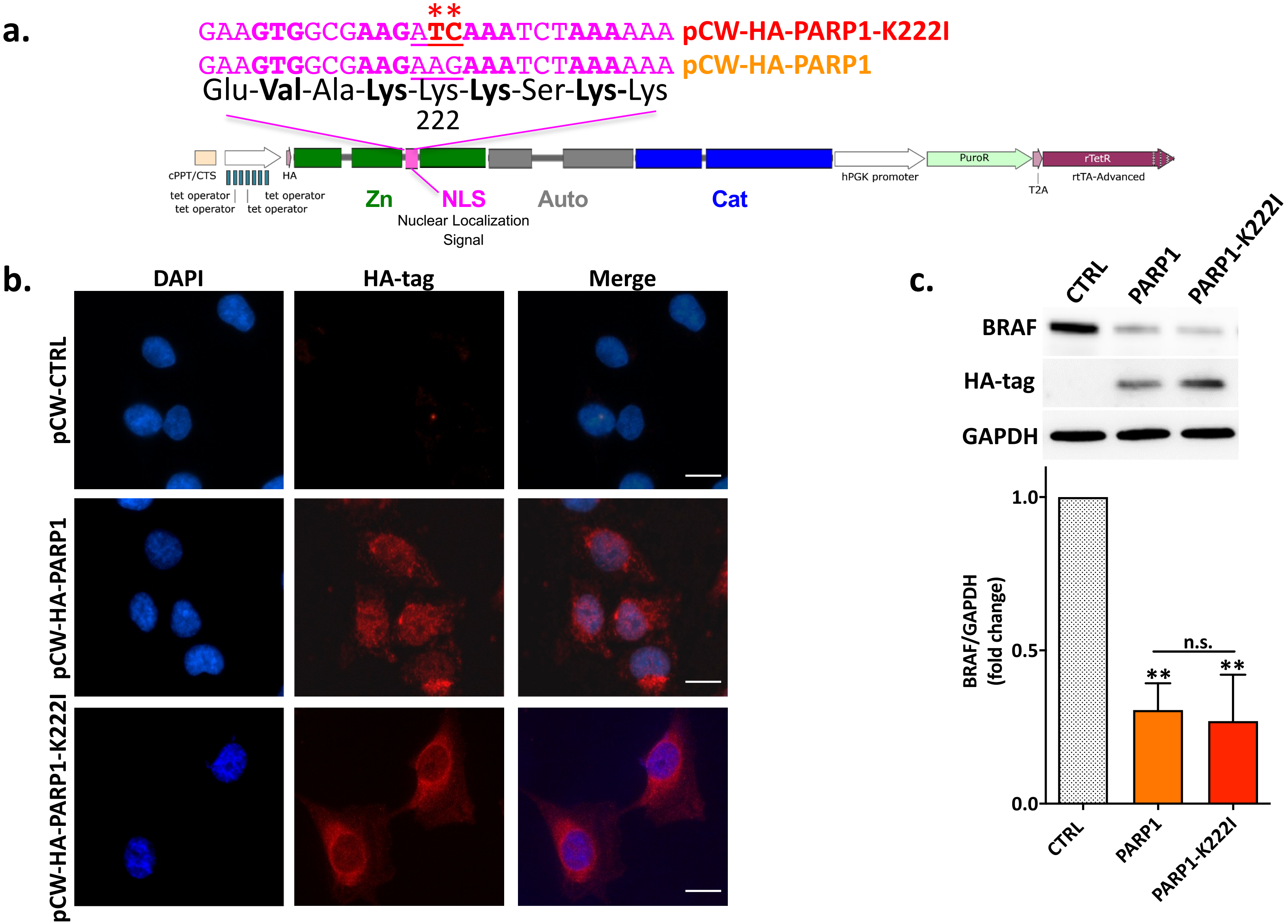

Supplementary Figure 14. The K222I mutant that is excluded from the nucleus can still downregulate endogenous BRAF protein level.

a. Schematic representation of the mutagenesis of residue 222 within the Nuclear Localization Signal (NLS) of PARP1. Lysine (K) is mutagenized into Isoleucine (I).

b. HA-tag detected by immunofluorescence in A375 cells stably infected with pCW-CTRL, pCW-HA-PARP1 or pCW-HA-PARP1-K222I, 48h after induction with 2ug/ml doxycycline. As reported in PMID: 30614530, the K222I mutation results in the exclusion of PARP1 from the nucleus. Blue: DAPI; red: HA-tag. Scale bar: 10um.

c. Western blot of A375 cells stably infected with pCW-CTRL, pCW-HA-PARP1 or pCW-HA-PARP1-K222I, 48h after induction with 2ug/ml doxycycline. A representative western blot result *(upper)* and bands quantification (mean±SEM of at least three independent experiments, *lower*) are shown. **p<0.01.

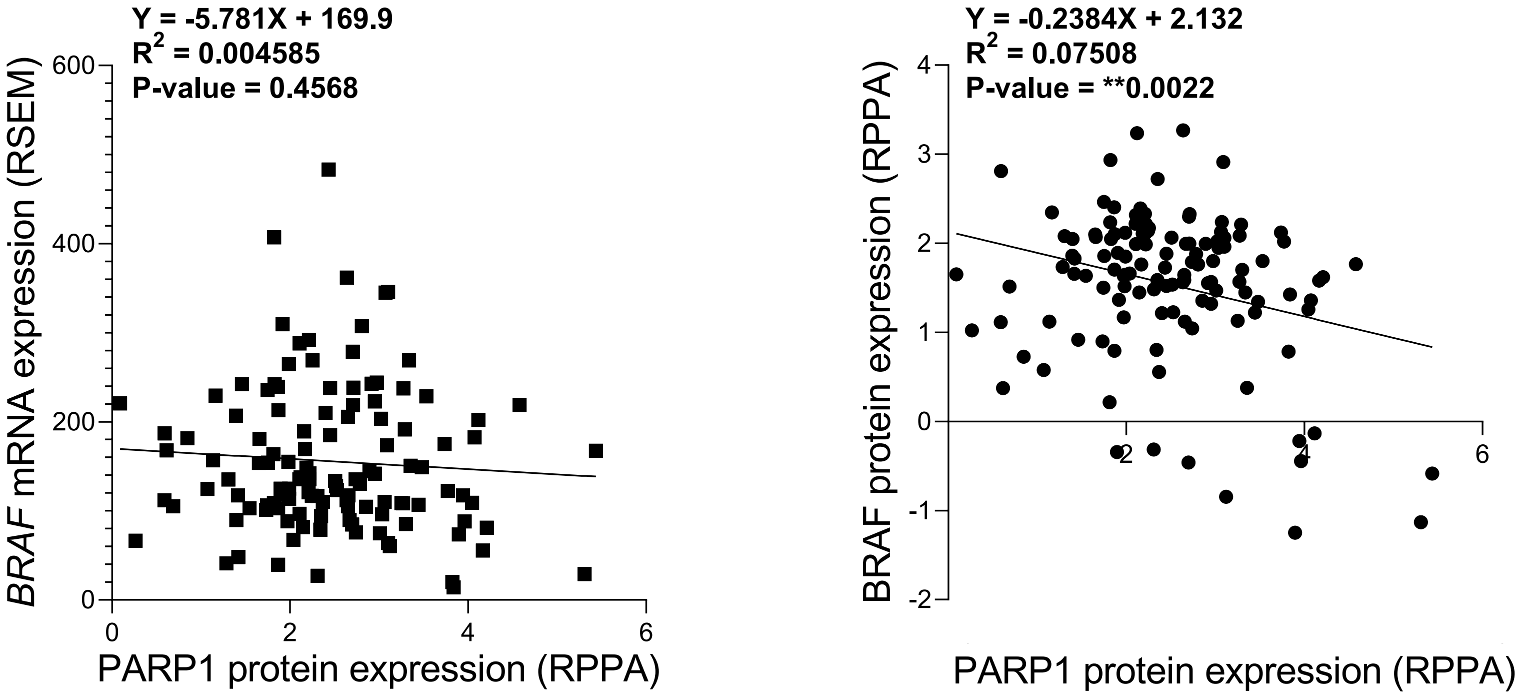

Supplementary Figure 15. Plot of *BRAF* mRNA level vs PARP1 protein level *(left)* and of BRAF protein level vs PARP1 protein level *(right)* within the TCGA-SKCM melanoma patient dataset.

Using the matched data of 122 melanoma patients, for which the levels of *BRAF* mRNA, BRAF protein and PARP1 protein are available on cBioportal website, we revealed the statistically significant negative correlation existing between BRAF protein level and PARP1 protein level. The subgroup of high PARP1 protein expressors (25% percentile, n=30) versus low PARP1 protein expressors (75% percentile, n=30) are analyzed in **Fig.1i,j** as well.

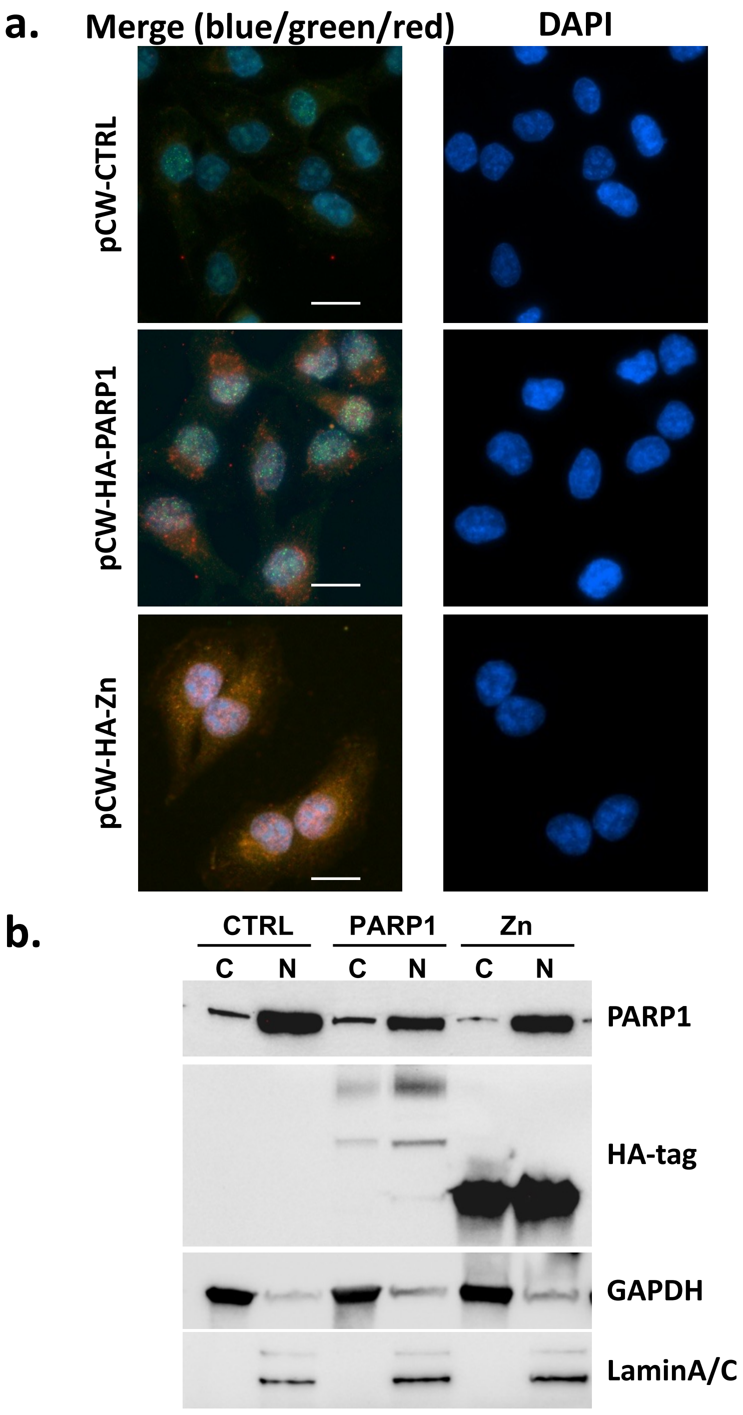

Supplementary Figure 16. The Zn domain has the same localization as full length PARP1.

a. Endogenous PARP1 and HA-tag detected by immunofluorescence in A375 cells stably infected with pCW-CTRL, pCW-HA-PARP1 or pCW-HA-Zn, 48h after induction with 2ug/ml doxycycline. Blue: DAPI; green: PARP1; red: HA-tag. Scale bar: 10um.

b. Western blot detection of PARP1 and HA-tag in the cytoplasmic (C) and nuclear (N) fractions obtained from A375 cells stably infected with pCW-CTRL, pCW-HA-PARP1 or pCW-HA-Zn, 48h after induction with 2ug/ml doxycycline. GAPDH and LaminA/C are included as positive controls for the cytoplasmic and nuclear fraction, respectively.

In overexpression, both full length PARP1 and the Zn domain localize in the nucleus and in the cytoplasm, like endogenous PARP1.

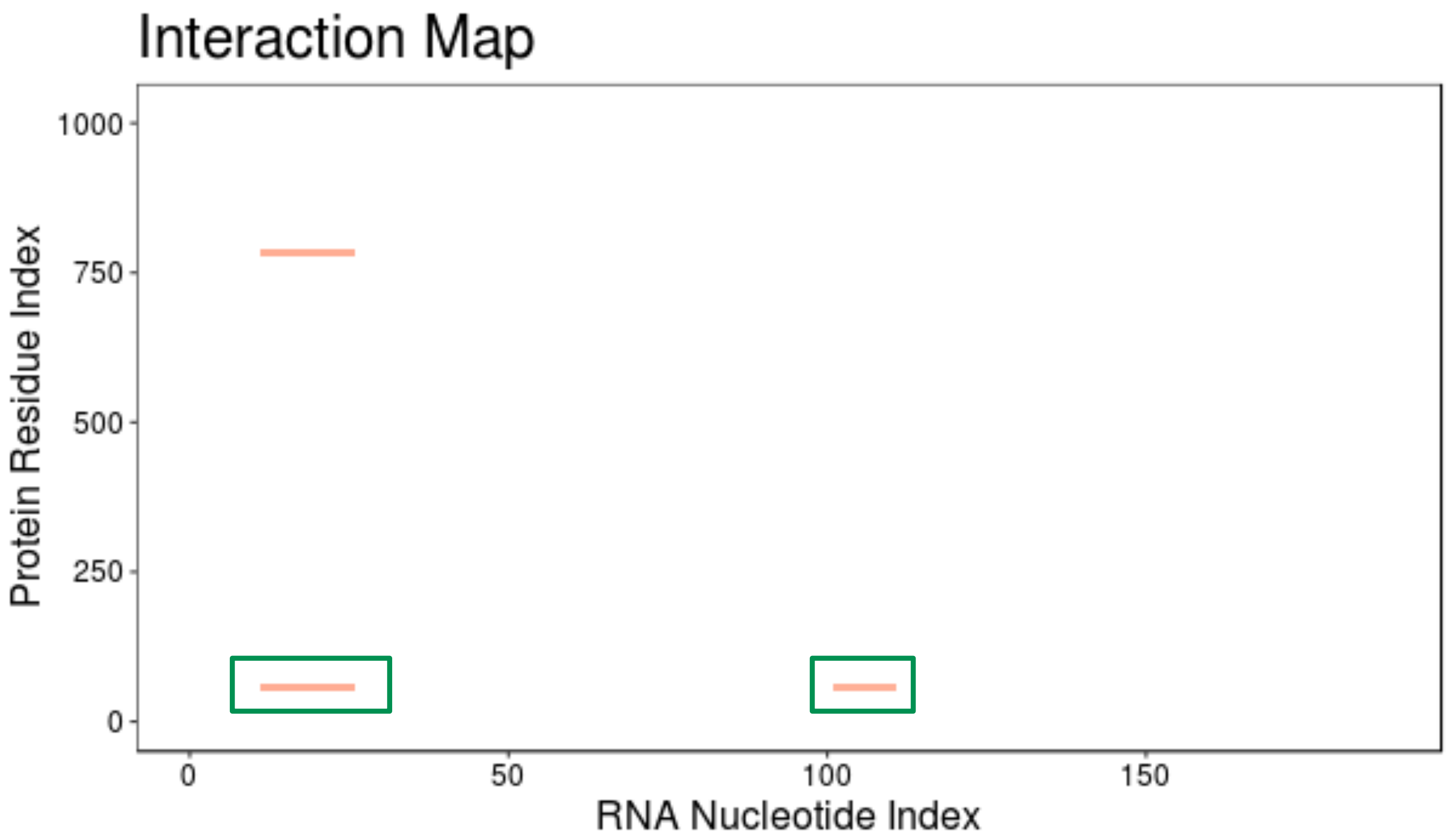

Supplementary Figure 17. Nucleotides of R8 fragment (x axis) and amino acids of PARP1 protein (y axis) involved in the interaction between the two molecules, as predicted by catRAPID omics v2.0 program.

Supplementary Table 4 ranks the binding affinity of the 20 selected mRBPs to the R8 fragment of *BRAF-X1 3'UTR*. The interaction map presented here reports the position of the top interaction sites of PARP1 with R8. Two interaction sites (green rectangles) are located between aa 1 - 250. This part of the protein corresponds to the Zn domain, which is known to bind nucleic acids (PMID: 34066057). The interaction between *X1* mRNA and PARP1 Zn domain was validated by us experimentally. A third interaction site is located between aa 750 – 1000. Within the Catalytic domain, these residues belong to the HD-ART motif, which is known to bind molecules like NADP+ and ADP+ (PMID: 34066057). This is likely the reason why the software algorithm, designed to find interactions between amino acids and nucleotides (PMID: 23264567), found this interaction site as well.

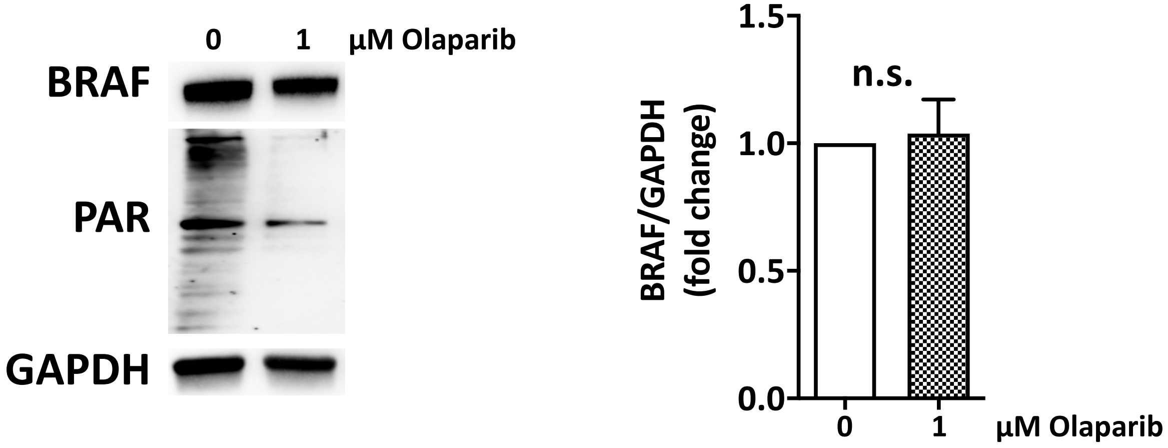

Supplementary Figure 18. Inhibition of parylation (PAR) activities by Olaparib does not affect BRAF protein level in A375 melanoma cells. Western blot *(left)* and bands quantification *(right)*. The graph represents the mean±SEM of at least three independent experiments.

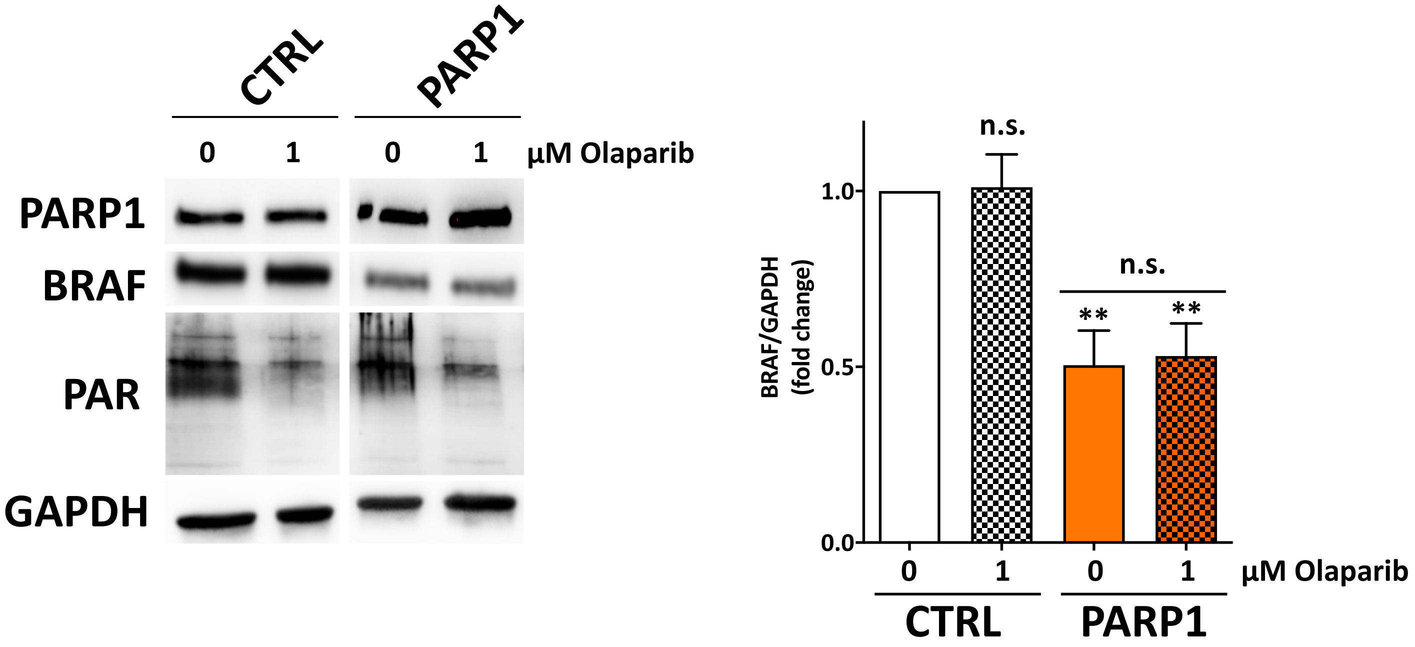

Supplementary Figure 19. PARP1 overexpression negatively regulates BRAF protein level, independently of its parylation (PAR) activities.
(left) Western blot of A375 cells stably infected with pCW-CTRL or pCW-PARP1, 48h after induction with 2ug/ml doxycycline and concomitant treatment with 1uM Olaparib. *(right)* Quantification of western blot bands. The graph represents the mean±SEM of at least three independent experiments. **p<0.01.

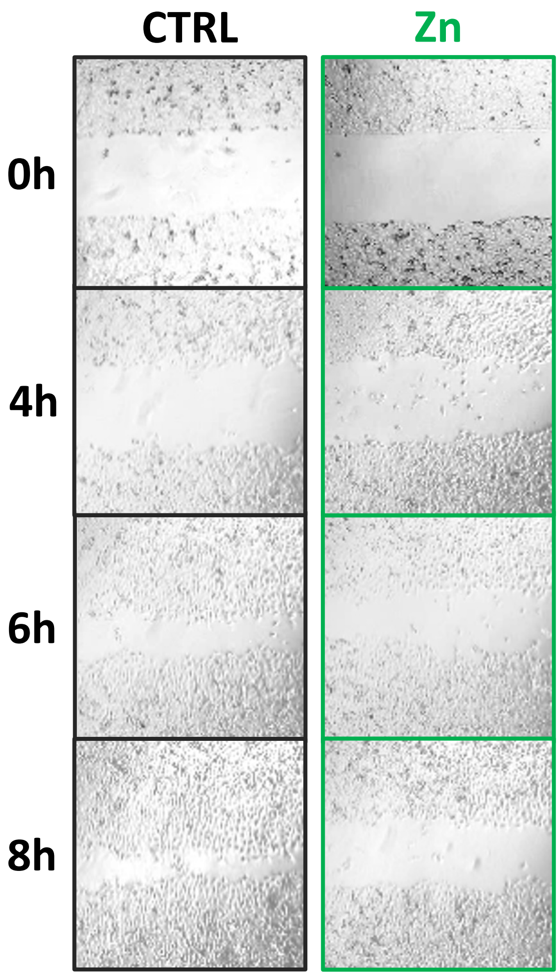

Supplementary Figure 20. Wound closure assay.
A375 cells, stably infected with pCW-CTRL or pCW-HA-Zn, were subjected to wound closure assay 48h after induction with 2ug/ml doxycycline. The quantification of relative wound closure is reported in **Fig.2i**.

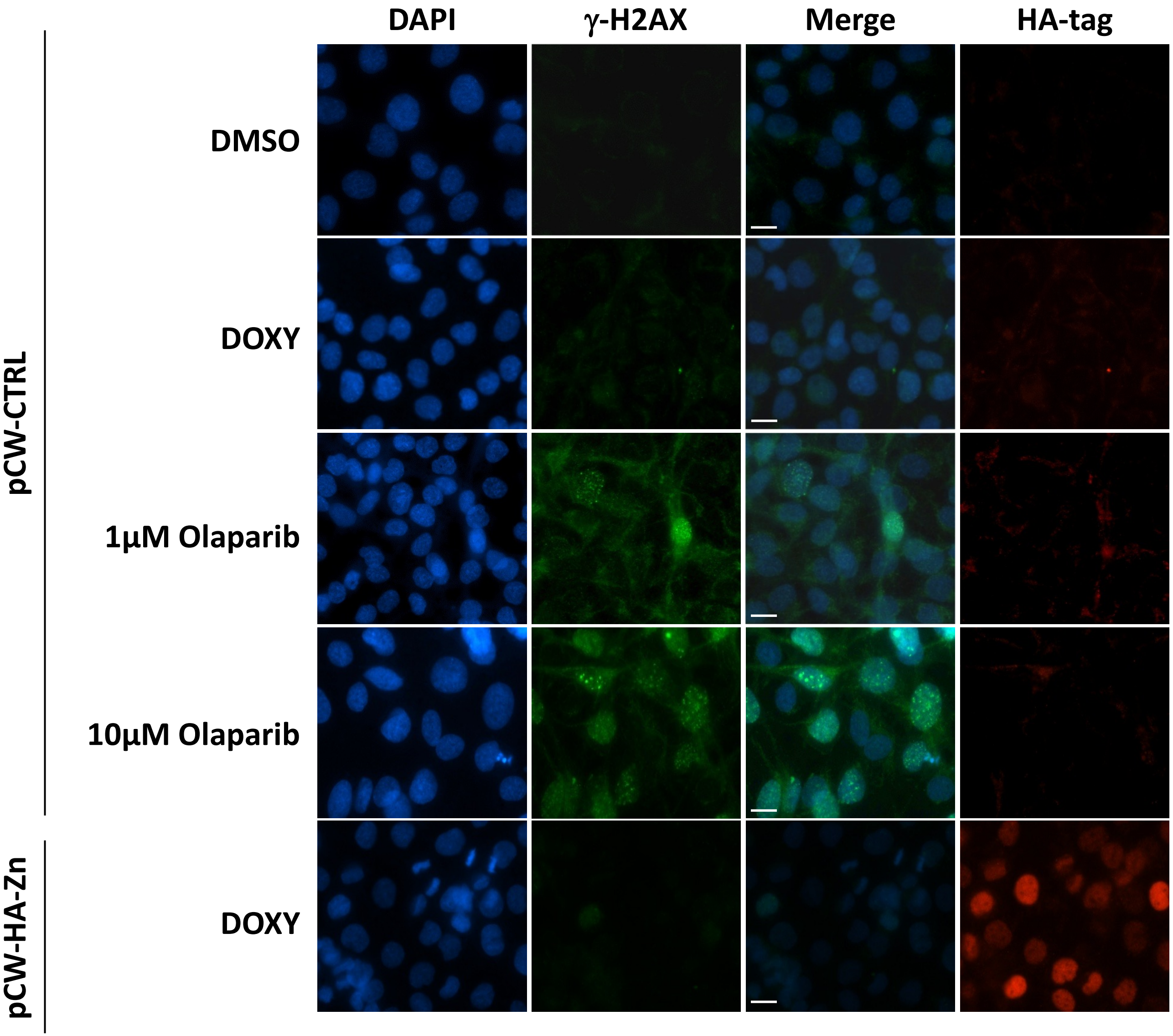

Supplementary Figure 21. Staining of γ-H2AX by immunofluorescence.

γ-H2AX foci were stained in A375 cells stably infected with pCW-CTRL or pCW-HA-ZnPARP1, 48h after induction with 2ug/ml doxycycline (DOXY). pCW-CTRL infected cells were concomitantly treated with the indicated concentrations of Olaparib. Blue: DAPI; green: γ-H2AX; red: HA-tag. Scale bar: 10um.
