# Supplementary material for: PARP1 negatively regulates MAPK signaling by impairing BRAF-X1 translation
Source: J Hematol Oncol. 2023 Apr 3;16:33. doi: 10.1186/s13045-023-01428-2 (PMC10071733; doi:10.1186/s13045-023-01428-2)
Supplement: Supplementary file 7 — Additional file 7. Material and Methods. [file 13045_2023_1428_MOESM7_ESM.docx]

**MATERIAL and METHODS**

**Primers and siRNAs**

All primers and siRNAs were purchased from Eurofins Genomics. Their sequence is reported in **Supplementary Table 1** and **Supplementary Fig.7**.

***In Vitro* transcription**

All DNA fragments used for riboprobe synthesis were obtained by PCR, using as template *BRAF-ref* *3’UTR* cloned in pGEM or *BRAF-X1* *3’UTR* cloned in pGEM [1]. The primers used for riboprobe synthesis are listed in **Supplementary Table 1**. The transcription reactions to obtain cold and ^[32P]^ UTP labelled probes were performed using the T7 in vitro transcription system MAXIScript T7 (Ambion).

**RNA electrophoretic mobility shift assay (REMSA)**

S100 cytoplasmic protein extracts were obtained from A375 cells lysed with five cell volumes of hypotonic buffer: 10mM HEPES pH 7.9, 1.5mM MgCl2, 10mM KCl, 0.2mM PMSF, 1xPI (protease inhibitors cocktail). The extracts were centrifuged at 1000xg and the supernatant was mixed with 0.11 volumes of S100 extraction buffer (0.3M HEPES pH 7.9; 30mM MgCl2; 1.4M KCl; 1xPI). After centrifugation at 100000g, the samples were dialysed against 50 volumes of dialysis buffer: 20mM HEPES pH 7.9, 10% glycerol, 40mM KCl, 3 mM MgCl2, 0.2mM PMSF. REMSA were performed as described previously [2]. In brief, 20ug of proteins were incubated for 30 min at room temperature with 1x10^5^ cpm of ^32P^-UTP-labelled riboprobes. After incubation, the reaction mixtures were treated with 10U RNase T1 to digest the RNA sequences unprotected by the complex binding and with heparin at a final concentration of 5 mg/ml, at 22°C. Competition assays were performed by mixing 20 and 200ng of cold riboprobes with labelled riboprobes in presence of the protein extract. The binding reactions were separated on native 6% PAGE and the gels were subsequently dried and radioactive image acquired by Typhoon analysis (Amersham Bioscience) [2]. The position of the bands is not indicative of the molecular weight of the protein complex.

**Pull-down of R8-binding RBPs**

To identify the proteins that bind R8 riboprobe, a pull-down experiment was performed. After *in vitro* synthesis, R8 riboprobe was biotinylated using Pierce RNA 3'-End Desthiobiotinylation Kit (Thermo Fisher Scientific) and incubated with 60ug of S100 cytoplasmic protein extract obtained from A375 cells. The R8-RBP complexes were then pulled down using PierceTM Magnetic RNA-Protein Pull-Down Kit (Thermo Fisher Scientific) according to the manufacturer’s instructions. The desthiobiotin tag binds to streptavidin magnetic beads in a manner that allows gentle elution of ribonucleoprotein complexes. To confirm the direct binding between desthiobiotinylated R8 riboprobe and PARP1, human recombinant PARP1 protein (#ALX-201-063, Enzo) was used in the pull-down assay. Desthiobiotinylated R2 riboprobe and desthiobiotinylated *3’UTR* of Androgen Receptor RNA (provided by the kit) were used as negative controls.

**LC-MS/MS-based Protein Identification**

Proteins bound to R8 and control samples (two biological replicates) were loaded onto a pre-cast NuPAGE 4-12% Bis-Tris mini gel (Invitrogen). The electrophoretic process was performed with MOPS buffer at 30mA and the run was stopped after about 15min, when all proteins were concentrated in a single band. The gel was stained with Coomassie G-250 (SimplyBlue SafeStain, Invitrogen), gel bands were excised, and proteins were in-gel digested with trypsin. Briefly, gel bands were cut in small pieces, dehydrated with acetonitrile (ACN) and dried under vacuum. The reduction of disulphide bridges was performed with 10mM dithiothreitol (DTT) in 50mM NH4HCO3 for 1h at 56°C and the alkylation of cysteines was carried out with 55mM iodoacetamide in 50mM NH_4_HCO_3_ for 45min at room temperature and in the dark. Proteins were digested with sequencing grade modified trypsin (12.5ng/ul in 50mM NH_4_HCO_3_, Promega) overnight at 37°C. Peptides were extracted from the gel by 3 changes of 50% ACN/0.1% formic acid (FA). Samples were dried under vacuum and suspended in 20ul of 3% ACN/0.1% FA. 8ul of each sample were analyzed with an LTQ-Orbitrap XL mass spectrometer (Thermo Fisher Scientific) coupled online with a nano-HPLC Ultimate 3000 (Dionex – Thermo Fisher Scientific). Peptides were separated with a linear gradient of ACN/0.1%FA from 3% to 40% in 40min and analyzed using a data dependent acquisition method: a full MS scan at high resolution in the orbitrap (60000 nominal resolution at 200 m/z) was followed by the MS/MS scan of the four most intense ions in the linear ion trap. A blank run was performed after each sample to reduce the impact of possible column carry-over.

Raw data files were analyzed with MaxQuant software package (version 1.5.1.2) [3] and MS/MS spectra were searched against the human section of the Uniprot database (version 20141201) with the following parameters: trypsin was selected as digesting enzyme with up to 2 missed cleavages allowed; precursor and fragment tolerance were set to 20 ppm and 0.5Da, respectively and carbamidomethyl cysteine and methionine oxidation were set as fixed and variable modifications, respectively. False discovery rate (FDR) was fixed at 0.01 at the protein, peptide and PSM level and proteins were grouped into protein families according to the principle of maximum parsimony. Data were filtered to remove contaminant proteins (such as keratins, trypsin, and streptavidin) and all protein groups that were identified with less than two unique peptides. The final list of R8 binding proteins was compiled by keeping only those proteins for which no peptides had been identified in the control group.

All parameters relevant to assess the quality of protein and peptide identifications and the robustness of protein quantification are reported in **Supplementary Table 2**.

**Plasmids**

pMIR-ref-3’UTR and pMIR-X1-3’UTR were previously described [4].

pMIR-X1-3’UTRΔR8. The *3’UTR* of *BRAF-X1*, deleted of the R8 portion, was amplified from pGEM BRAF-X1 3’UTR plasmid [1], with Phusion Flash High-Fidelity Master mix (Thermo Fisher Scientific), using X1-ΔR8-SpeI-F and X1-ΔR8-HindIII-R primers **(Supplementary Table 1**). The PCR product was subsequently cloned downstream of the Firefly Luciferase CDS in the pMIR-REPORT miRNA Expression Reporter Vector (pMIR for brevity, Thermo Fisher Scientific), using SpeI and HindIII restriction enzymes.

pTK. This plasmid expresses the Renilla Luciferase and is cotransfected with pMIR plasmid as normalization control [1].

pCW-PARP1. The CDS of PARP1 was amplified from pYES2-PARP1 plasmid (kind gift from Dr. A. Galli, CNR, Pisa, Italy) with Phusion Flash High-Fidelity Master mix (Thermo Fisher Scientific), using the primers listed in **Supplementary Table 1**. The PCR product was subsequently cloned downstream of the Tet operator in the pCW vector [4], using NheI and MluI restriction enzymes.

pCW-HA-PARP1, pCW-HA-Zn, pCW-HA-Auto and pCW-HA-Cat. First, pCW-HA was created, by cutting pCW vector with NheI and SalI restriction enzymes, and by cloning a double strand oligo that contains the HA-tag sequence. To obtain such oligo, HA_Top_Strand and HA_Bottom_Strand oligos (**Supplementary Table 1**) were previously annealed and phosphorylated at 5’ends using PNK enzyme.

Then, full length PARP1, as well as Zn, Auto and Cat domains, were amplified from pYES2-PARP1 plasmid (see above) with Phusion Flash High-Fidelity Master mix (Thermo Fisher Scientific), using the primers listed in **Supplementary Table 1**. For subsequent ligations, PCR products were cloned into pCW-HA vector using appropriate restriction enzymes (SpeI and SalI for PCR products, NheI and SalI for pCW-HA vector).

pCW-HA-PARP1-K222I. The mutation K222I (AAG (K-Lys)🡪**ATC** (I-Ile)) within the NLS of PARP1 CDS was introduced in pCW-HA-PARP1 vector, using Phusion Flash High-Fidelity Master mix (Thermo Fisher Scientific) and NLS_mut_K221I_F/R primers (**Supplementary Table 1**). The PCR product was digested with DpnI enzyme to remove template DNA and it was subsequently transformed into competent bacterial cells. Following sequencing, the mutant insert was amplified using PARP1-Zn-F-SpeI and PARP1-Cat-R-MluI primers, digested with SpeI and MluI restriction enzymes and re-cloned into pCW-HA vector, previously digested with NheI and MluI restriction enzymes.

All restriction and modification enzymes used for cloning were purchased from New England Biolabs.

pCW vector maps are reported in **Supplementary Fig.9,13,14**. The empty pCW vector was used as negative control for all infections (pCW-CTRL).

**Cell Culturing**

A375, A375 C2 and 501Mel cell lines were cultured using DMEM high glucose supplemented with 10% fetal bovine serum, 1% glutamine and 1% penicillin/streptomycin, as previously described in [4,5].

**Transfection of siRNAs**

3x10^5^ melanoma cells were transfected in suspension in 6-well plates. 2.5ul of 20uM siRNA stock solution were added to 100ul OptiMEM I® (Thermo Fisher Scientific), while 4ul of 1mg/ml LIPOFECTAMINE 2000™ (Thermo Fisher Scientific) were added to additional 100ul of OptiMEM I®. These two solutions were then combined and the siRNA-LIPOFECTAMINE 2000™ complexes were allowed to form for 15min at room temperature, following the manufacturer’s instructions. In the meantime, cells to be transfected were resuspended in 800ul of OptiMEM I® medium (3.75x10^5^ cells/ml final concentration). The siRNA/LIPOFECTAMINE 2000™ mixture was added to the wells and then the 800ul of cell suspension were added to the same well and mixed.

After 6h, medium was replaced with complete medium for 24/48h, in order to perform qRT-PCR/western blot analyses. In alternative, at the end of the 6 hours, cells were trypsinized and were used for cellular assays.

**Lentiviral transduction**

Stable infection of melanoma cell lines with pCW-CTRL, pCW-PARP1, pCW-HA-PARP1, pCW-HA-PARP1-K222I, pCW-HA-Zn, pCW-HA-Auto and pCW-HA-Cat lentiviral vectors was performed as described in [4]. Briefly, pCW vectors were cotransfected in HEK293 cells with 2 plasmids encoding proteins necessary for virus packaging. After 48h, media containing lentivirus particles were harvested and viruses were pulled down by using Lenti-X^TM^ Concentrator (TaKaRa), following the manufacturer’s protocol. Melanoma cells were then transduced with lentiviruses plus polybrene (4ug/ml). Pools of stably transduced cells were selected by adding puromycin (2ug/ml) to the culture medium. 2ug/ml doxycycline was used in the experiments to induce transgene expression.

**Proliferation assay**

This assay was performed as described previously [4]. Briefly, 1-2x10^3^ stably infected cells were seeded in 12-well plates (3 wells per experimental condition). 24h later, they were treated with 2ug/ml doxycycline to induce transgene expression, and with the appropriate dose of drug or vehicle (DMSO) if needed. 7-14 days later, cells were fixed with 4% PFA and stained using a crystal violet solution (0.1% crystal violet, 20% methanol, in water). After staining, the excess of crystal violet was washed with tap water and plates were dried. Cells were de-stained using a 10% acetic acid solution and the absorbance of the solution was then measured at 590nm.

**Wound closure assay**

2.5×10^4^ stably infected cells were seeded in silicone inserts (Culture-Insert Family, IBIDI). 24h later (t0), when ~70–80% confluence was reached, the inserts were removed and the quality of the covered surface was evaluated. Then, 2ug/ml of doxycycline were added to the media. The cell-free gap was measured at different time points after insert removal plus induction, using 10× and 20× objective lens (Leica DM IL LED microscope). Image J software (<http://rsb.info.nih.gov>) was used to measure the cell-free gap area and the migratory rate was determined as percentage of gap closure compared to the t0 area. For each condition, ‘wounds’ from three independent experiments were measured.

**Total ROS analysis**

Stably infected cells were plated in 12-well plates and the day after they were treated for 48h with 2ug/ml doxycycline (for transgene induction), as well as with 2uM vem or vehicle (DMSO). Then, total ROS were analyzed using CellROX Deep Red Reagent (C10422, Thermo Fisher Scientific). According to the manufacturer’s instructions, cells were incubated for 30min with CellROX reagent, then washed three times with PBS. For each sample, 10.000 events were analyzed by flow cytometry (C6 Accuri, BD).

**RNA extraction and DNAse treatment**

RNA was extracted using QIAzol reagent (QIAGEN), following the manufacturer’s instructions and it was subsequently quantified using Nanodrop Lite (Thermo Fisher Scientific). RNA purity was assessed by checking the A260/A280 ratio, while its integrity was assessed by agarose gel electrophoresis. Then, 1ug RNA/10ul was subjected to DNAse I treatment (Thermo Fisher Scientific), following the manufacturer’s protocol.

**Retrotranscription and real-time PCR (qRT-PCR)**

250ng of RNA were retrotranscribed in a 10ul reaction using iSCRIPT RT supermix (Bio-Rad), following the manufacturer’s instructions. To exclude genomic contamination, a PCR is performed using ATPA1 primers (ATPA1-F CTCAGATGTGTCCAAGCAAG and ATPA1-R GTCAGTGCCCAAGTCAATG). These primers produce a genomic-derived amplicon of 300bp and a cDNA derived amplicon of 180bp, as described in [6]. Real-time PCR (qRT-PCR) was performed in triplicate, using 2ul of a 1:4 dilution of cDNA, appropriate qRT-PCR primers (0.5uM each, **Supplementary Table 1**) and SSOADV Universal SYBR green (Bio-Rad) in 15ul final reaction volume (see also [2]). qRT-PCR reactions were performed in a CFX96 Real-Time System (Bio-Rad) using the following amplification conditions: 30s 98°C (3s 98°C, 20s 58-60°C, 10s 72°C)x40 cycles. Melting curve analysis and agarose gel electrophoresis of the PCR products were performed to assess the specificity of the reaction. qRT-PCR primers were designed to be exon spanning (when possible) and to produce 90-110bp long amplicons. Relative expression of targets was determined using the 2^-∆∆Ct^ method and data were normalized using 3 housekeeping genes (*GAPDH*, *HMBS* and *SDHA*).

**Actinomycin D assay**

Cells were transfected with the siRNA in 6-well plates, as described above (2 wells for each siRNA). The day after transfection, cells were treated with 10ug/ml Actinomycin D (Sigma-Aldrich) or with DMSO for 8h. Total RNA was then extracted, and qRT-PCR performed.

**Dual Luciferase reporter assay**

1.5x10^5^ cells per 24-well plate were transfected in suspension by using LIPOFECTAMINE 2000™ (Invitrogen).

50ng of pMIR-ref-3’UTR, pMIR-X1-3’UTR or pMIR-X1-3’UTRΔR8 plasmid and 10ng of pTK plasmid were added to 50ul OptiMEM I® (Invitrogen), while 2.5ul of 1mg/ml LIPOFECTAMINE 2000™ (Invitrogen) were added to additional 50ul of OptiMEM I®. The two solutions were then combined, and the mixture was let stand for 15min at room temperature. In the meantime, cells to be transfected were resuspended in 400ul of OptiMEM I® medium (3.75x10^5^ cells/ml final concentration). The plasmid/LIPOFECTAMINE 2000™ mixture was added to the wells and then the 400ul of cell suspension were added to the same well and mixed.

After 6h, transfection medium was replaced with complete medium. 48h after transfection, cells were lysed and Luciferase activity was measured with GloMax®-Multi Microplate Multimode Reader (Promega) luminometer, using the Dual-Luciferase® Reporter Assay kit (Promega) and following the manufacturer’s instructions.

If siRNAs were used, 1.5ul of 20uM siRNA stock solution were added to the 50ul of OptiMEM I® containing pMIR and pTK plasmids.

If stably infected cells were used, transfection medium was replaced with complete medium plus 2ug/ml doxycycline.

**Relative Translational Efficiency (RTE) assay**

This assay was performed as described in [4]. Briefly, cells were transfected in triplicate as described above in “Dual Luciferase reporter assay”. After 6h, medium was replaced with fresh complete medium, supplemented with 2ug/ml doxycycline if needed. 48h after transfection, 2 wells were used to detect Luciferase protein activity, while the other one was harvested and subjected to RNA extraction, retrotranscription and qRT-PCR to measure Luciferase mRNA level. The Relative Translation Efficiency (RTE) was then calculated using the following formula:

RTE = [RLU_(x)_ ^FLuc/RLuc^ / RNA_(x)_ ^FLuc/RLuc^] / [RLU_CTRL_ ^FLuc/RLuc^ / RNA_CTRL_ ^FLuc/RLuc^].

RLU: Relative Luciferase Units; FLuc: Firefly Luciferase; RLuc: Renilla Luciferase.

**Western blot**

Proteins were extracted from cells by 5 mM Tris pH8, 1% TritonX 100, 0.25% Sodium Deoxycholate, 10% proteinase inhibitor, 2% PMSF, 0.5% Ortovanadate. The samples were then heated at 95 °C for 5 min, separated on 10% SDS-polyacrylamide gels (Mini-PROTEAN Precast gel, Bio-Rad) and electrotransferred to polyvinylidene difluoride (PVDF) membranes using Trans-Blot Turbo system (Bio-Rad) as previously reported in [4]. Proteins were detected by the following primary antibodies:

- anti-LaminA/C (mab636, # MA3-1000, Thermo Fisher Scientific; mouse monoclonal antibody, dilution 1: 2000 in 3% milk in TBST);
- anti-BRAFV600E (VE1, #ab228461, Abcam; mouse monoclonal antibody, dilution 1:400 in 3% milk in TBST);
- anti-BRAF-X1 (rabbit polyclonal antibody, dilution 1:1000 in 3% milk in TBST). This custom antibody was developed at Moravian Biotechnology (http://www.moravian-biotech.com), using the X1-specific C terminal peptide (CGGYGEFAAFK) as immunogen [4];
- anti-GAPDH (14C10, #2118, Cell Signaling Technology; rabbit monoclonal antibody, dilution 1:3000 in 3% milk in TBST);
- anti-HA-tag (C29F4, #3724, Cell Signaling Technology; rabbit monoclonal antibody, dilution 1:2000 in 3% milk in TBST);
- anti-MEK 1/2 (L38C12, #4694, Cell Signaling Technology; mouse monoclonal antibody, dilution 1:1000 in 1% BSA in TBST);
- anti-PAR (#4336-BPC-100, Trevigen; rabbit polyclonal antibody, dilution 1:1000 in 3% milk in TBST);
- anti-PARP1 (F-2, #sc-8007, Santa Cruz Biotechnology; mouse monoclonal antibody, dilution 1:1000 in 3% milk in TBST).
- anti-pMEK (41G9, #9154, Cell Signaling Technology; rabbit monoclonal antibody, dilution 1:1000 in 1% BSA in TBST).

**Fractionation**

Nuclear and cytoplasmic fractions were obtained from 2x10^6^ cells by NE-PER™ Nuclear and Cytoplasmic Extraction Reagents (Thermo Fisher Scientific), according to manufacturer’s instructions. Nuclear and cytoplasmic fractions were then analyzed by western as described above.

**Immunofluorescence microscopy and semi-quantification of γ-H2AX foci**

5x10^4^ cells were seeded on glass cover slips in a 12-well plate and treated for 48h with doxycycline. Then they were fixed with 4% formaldehyde for 10 min. Before adding primary and secondary antibodies, cells were permeabilized twice for 10min with 0.1% Triton in PBS and washed with blocking solution (0.5% BSA/0.15% glycine in PBS). Anti-PARP1 (F-2, #sc-8007, Santa Cruz Biotechnology; mouse monoclonal antibody, dilution 1:500 in blocking solution), anti-γ-H2AX (phospho-Histone H2AX (Ser139), JBW301, #05-636-I, Millipore; mouse monoclonal antibody, dilution 1:200 in blocking solution) and anti-HA-tag (C29F4, #3724, Cell Signaling Technology; rabbit monoclonal antibody, dilution 1:800 in blocking solution) were incubated at room temperature for 1h. Then, anti-mouse (#A-11001, Invitrogen) or anti-rabbit (#A-11037, Invitrogen) secondary antibodies were applied, diluted 1:500 in blocking solution containing 50ng/ul DAPI, for 1h at room temperature. Finally, the cells were washed and Fluoromount^TM^ (K024 Diagnostic BioSystems) was used as a mounting reagent.

**Fluorescence microscopy and analysis of foci formation**

Analysis of foci formation was performed using Nikon ECLIPSE Ti2-E inverted fluorescence microscope equipped with a 60X objective. The number of cells containing foci was determined by counting 150–300 cells per experiment.

**RNA immunoprecipitation coupled with qRT-PCR quantification (RIP-qRT-PCR)**

2x10^6^ stably infected cells were seeded in a 10cm dish and were treated with 2uM doxycycline for 48h. Cells were then harvested and lysed with 600ul of complete Polysome Buffer (20mM Hepes, pH 7.5, 100 mM KCl, 5mM MgCl 2, 0.5% NP-40, protease inhibitors (Roche), 100U/ml RNAse OUT (Invitrogen), and 10mM dithiothreitol (DTT)) for 5 min on ice. The lysate was clarified by centrifugation at 15000g for 15 minutes at 4°C. 500ul of the supernatant were used for RIP and 100ul were saved for subsequent RNA purification (INPUT). For each sample of the RIP, 25ul of anti-HA-tag sepharose beads (C29F4, #3956, Cell Signaling Technology) were washed tree times with 1ml of NT2 buffer (50mM Tris-HCl at pH 7.5, 150mM NaCl, 1mM MgCl_2_ and 0.05% NP-40) and were resuspended in 50ul of the same buffer supplemented with 100U/ml RNase OUT, 1mM DTT and 17.5uM EDTA. The beads were then added to the lysate and incubated at 4°C overnight on a rotating shaker. After incubation, the beads were washed 5 times with 1ml of ice-cold NT2 buffer, by pulsing them in a centrifuge and removing the supernatant with a pipette. After the last wash, 700ul of Qiazol (QIAGEN) were added to the bead pellet and RNA was extracted using miRNeasy mini kit (QIAGEN) following the manufacturer’s instructions. Extracted RNA was subjected to qRT-PCR (see above), using BRAF-X1-638/732, BRAF-X1-754/861, BRAF-ref and HMBS primers.

**STRING functional enrichment and Cytoscape network**

The Protein Interaction Network of the 51 proteins that were prioritized among the 87 identified by mass spectrometry was made using stringApp v1.7.0, on the Cytoscape Network Data Integration, Analysis, and Visualization Software [7]. The protein network was imported from STRING v11.5 [8], while functional enrichment was made thanks to STRING Application Programming Interface, which provides raw p-values, False Discovery Rate and Bonferroni corrected p-values. The default settings of the STRING enrichment were as follows: number of terms to chart: 5; overlap cutoff: 0.5.

**Prediction of protein-RNA interaction**

The prediction of the interaction between the pool of 20 selected mRBPs and R8 RNA fragment was calculated thanks to catRAPID omics v2.0 program, a freely accessible web server for the prediction of protein-RNA interactions [9]. The input sequences were set in FASTA format. In turn, FASTA sequences were downloaded from The Universal Protein Resource (UniProt) database. Rank value indicates the propensity of R8 to bind a certain protein in the list.

**Visual representation of the PARP1–R8 complex**

The structural model of human PARP1 (PDB: AF-P09874-F1-model_v1) was retrieved from AlphaFold Protein Structure Database [10] and used as starting coordinates for the docking calculations. Analogously, the structural model of the R8 RNA fragment was generated thanks to the HDOCK server for protein-DNA/RNA docking and template-based modeling [11].

The docking procedure was done through the HDOCK webserver as well. In the docking method, a standard protocol was used and an ensemble of one hundred poses was generated for each docking run. The receptor/RNA complex was selected from the ensemble and used as visual representation of the interaction between PARP1 protein and R8 RNA.

The visual representation and the graphics of the complex were curated using the UCSF ChimeraX software (https://www.cgl.ucsf.edu/chimerax/).

**TCGA analysis**

Clinical data, RNA expression data and protein expression data of TCGA-SKCM patients were downloaded from <http://gepia.cancer-pku.cn/detail> or <https://www.cbioportal.org/>, and statistical analyses were performed by GraphPad Prism (GraphPad Software Inc.).

**Xenograft in zebrafish embryos**

Xenograft assays were performed as described previously [4,5]. Briefly, 48h before injection in embryos, 5x10^5^ cells were seeded in 100mm plates (1 plate per experimental condition).

After 48h, cells were harvested using trypsin, counted, centrifuged (5min 300xg) and washed once with PBS. Finally, they were resuspended in 5ml of PBS solution containing 5uL of C7001 Cell Dye (Invitrogen, 1 mg/ml in DMSO stock solution) and incubated 15min at 37°C, then 15min at 4°C.

Stained cells were centrifuged again (5min 300xg) and were resuspended with 1ml of PBS. Cell suspensions were transferred in a 1.5ml tube and subjected to an additional centrifugation step (5min 300xg). PBS was completely removed, and the tube was cooled down on ice. For tumor growth analysis, cells were carefully resuspended in matrigel (Cultrex Basement Membrane Extract, PathClear) to reach the final concentration of 250 cells/nl (4ul of matrigel per 1x10^6^ cells), by using cold pipette tips and avoiding bubble formation. For migration assays, cells were washed once in FBS and then in PBS. Cells were finally resuspended with PBS at 200cells/nl (5ul per 1x10^6^ cells) final concentration.

Concurrently to cell staining procedures, 48hpf zebrafish embryos of the *Tg(kdrl:EGFP)* strain (kindly provided by Dr. Massimo Santoro, University of Padua, Italy) were dechorionated manually by forceps (Dumont No. 5, #F6521-1EA, Sigma-Aldrich) and anesthetized with 0.17mg/ml tricaine (Sigma-Aldrich, A5040). Cell suspension was loaded into a borosilicate glass capillary and 1nl (250 cells for proliferation or 200 cells for migration) were injected into the yolk sac of the dechorionated embryos, using a microinjector (Tritech Research). 3h post injection, embryos were treated with 2uM vem or vehicle (DMSO) and with 2ug/ml doxycycline in E3 medium (5mM NaCl, 0.17mM KCl, 0.33mM CaCl_2_, 0.33mM MgSO_4_). Then, they were incubated at 36°C for 48/96h (authorization #383/2020-PR). At least 80 embryos were injected per experimental condition and each experiment was repeated three times. At the end of treatment period, fluorescence imaging was carried out using the Nikon Eclipse E600 microscope equipped with CoolSnap-CF camera and NIS-Elements software version 4.0. Pictures of the tumor xenograft were acquired soon after microinjection and after 48h of treatment. For migration analysis, pictures were taken after microinjection and after 96h of treatment. Tumor area, as well as metastases size and distance from injection site, were measured using ImageJ software (http://rsb.info.nih.gov). Data were analyzed using non-parametric Kruskal-Wallis test (Dunn's multiple comparisons test) (GraphPad Prism, GraphPad Software Inc.).

**Statistical analyses**

Unless specified otherwise, data of at least 3 independent experiments were analyzed using unpaired and two-tailed t test (GraphPad Prism, GraphPad Software Inc.). Values of p < 0.05 were considered statistically significant (*p < 0.05, **p < 0.01, *** < 0.001, ****p < 0.0001).

**REFERENCES**

1. Marranci A, Tuccoli A, Vitiello M, Mercoledi E, Sarti S, Lubrano S, et al. Identification of BRAF 3’UTR isoforms in melanoma. Journal of Investigative Dermatology. 2015;135.

2. Corso C, Pisapia L, Citro A, Cicatiello V, Barba P, Cigliano L, et al. EBP1 and DRBP76/NF90 binding proteins are included in the major histocompatibility complex class II RNA operon. Nucleic Acids Res. 2011;39:7263–75.

3. Tyanova S, Temu T, Cox J. The MaxQuant computational platform for mass spectrometry-based shotgun proteomics. Nat Protoc. England; 2016;11:2301–19.

4. Marranci A, D’Aurizio R, Vencken S, Mero S, Guzzolino E, Rizzo M, et al. Systematic evaluation of the microRNAome through miR-CATCHv2.0 identifies positive and negative regulators of BRAF-X1 mRNA. RNA Biol. 2019;16:865–78.

5. Marranci A, Jiang Z, Vitiello M, Guzzolino E, Comelli L, Sarti S, et al. The landscape of BRAF transcript and protein variants in human cancer. Mol Cancer. 2017;

6. Vitiello M, Tuccoli A, D’Aurizio R, Sarti S, Giannecchini L, Lubrano S, et al. Context-dependent miR-204 and miR-211 affect the biological properties of amelanotic and melanotic melanoma cells. Oncotarget. 2017;8:25395–417.

7. Shannon P, Markiel A, Ozier O, Baliga NS, Wang JT, Ramage D, et al. Cytoscape: a software environment for integrated models of biomolecular interaction networks. Genome Res. 2003;13:2498–504.

8. Szklarczyk D, Gable AL, Nastou KC, Lyon D, Kirsch R, Pyysalo S, et al. The STRING database in 2021: customizable protein-protein networks, and functional characterization of user-uploaded gene/measurement sets. Nucleic Acids Res. 2021;49:D605–12.

9. Agostini F, Zanzoni A, Klus P, Marchese D, Cirillo D, Tartaglia GG. catRAPID omics: a web server for large-scale prediction of protein-RNA interactions. Bioinformatics. 2013;29:2928–30.

10. Jumper J, Evans R, Pritzel A, Green T, Figurnov M, Ronneberger O, et al. Highly accurate protein structure prediction with AlphaFold. Nature. 2021;596:583–9.

11. Yan Y, Tao H, He J, Huang S-Y. The HDOCK server for integrated protein-protein docking. Nat Protoc. England; 2020;15:1829–52.
